# Environmental noise-induced changes to the IC-SNc circuit promotes motor deficits and neuronal vulnerability in a mouse model of Parkinson’s Disease

**DOI:** 10.1371/journal.pbio.3003435

**Published:** 2025-11-04

**Authors:** Chi Cui, Yibo Yao, Yulong Shi, Jie Lei, Kun Ren, Kexing Wan, Tongxia Li, Gangan Luo, Qian Xu, Ming Li, Xiang Peng, Xueke Yang, Jian Yang, Junsong Du, Sitong Chen, Bo Tian, Pei Zhang

**Affiliations:** 1 Hubei Provincial Clinical Research Center for Alzheimer’s Disease, Tianyou Hospital, School of Medicine, Wuhan University of Science and Technology, Wuhan, Hubei, China; 2 Department of Neurobiology, School of Basic Medicine, Tongji Medical College, Huazhong University of Science and Technology, Wuhan, Hubei, China; 3 School of Clinical Traditional Chinese Medicine, Hubei University of Chinese Medicine, Wuhan, Hubei, China; 4 Institute for Brain Research, Huazhong University of Science and Technology, Wuhan, Hubei, China; 5 Key Laboratory of Neurological Diseases, Ministry of Education, Wuhan, Hubei, China; Northwestern University, UNITED STATES OF AMERICA

## Abstract

Emerging clinical evidence suggests a link between environmental noise and the severity of Parkinson’s disease (PD). However, the effects of high-decibel noise exposure on PD and its underlying mechanisms remain unclear. In this study, we demonstrate that acute noise exposure induces reversible motor deficits in subacute low-dose 6-hydroxydopamine (6-OHDA) mice, a model of presymptomatic early-stage PD, while chronic noise exposure results in irreversible motor deficits and significant loss of substantia nigra compacta (SNc) dopaminergic (DA) neurons. Additionally, noise exposure activates the inferior colliculus (IC), which sends monosynaptic projections to SNc^DA^ neurons. Optogenetic or chemogenetic bidirectional activation or inhibition of the IC-SNc circuit can mimic or reverse the 6-OHDA vulnerability caused by acute or chronic noise exposure. Mechanistically, noise exposure and IC-SNc circuit activation down-regulate vesicular monoamine transporter 2 (VMAT2) in the SNc, and overexpression of VMAT2 in IC-innervated SNc^DA^ neurons ameliorates noise exposure-induced 6-OHDA vulnerability. Our findings uncover a previously unappreciated role of the IC-SNc circuit in early-stage PD mice in response to environmental noise, which has significance for preventing the onset and progression of PD and highlights the need for environmental harmony to reduce neurodegeneration.

## Abstract 2

Prolonged exposure to environmental noise has been associated with the occurrence neurodegenerative diseases. This study demonstrates that environmental noise promotes the onset of Parkinson's disease phenotypes in a mouse model and shows that the IC-SNc circuit mediates this increased susceptibility.

## Introduction

Parkinson’s disease (PD) is a secondary degenerative disorder of the central nervous system characterized by prominent motor symptoms such as tremors, bradykinesia, muscle rigidity, and postural instability [1]. As the condition advances, PD patients may also manifest a spectrum of non-motor symptoms encompassing cognitive, psychiatric, and autonomic dysfunctions [2]. The etiology and progression of PD are multifactorial, involving genetic predisposition as well as environmental and lifestyle influences [3–6]. Our previous research has established the key role of genetic factors in PD [7–9]. However, the impact of environmental factors, particularly environmental noise, on PD is still largely unknown.

Environmental noise is widespread in modern urban and industrialized societies and has been associated with significant negative effects on both auditory and non-auditory systems in observational studies [10,11]. There is evidence to suggest that rodents exposed to high noise levels (>100 decibels [dB] sound pressure level [SPL]) not only damage the auditory peripheral, but also weaken central auditory processing, leading to auditory disorders [12,13]. While moderate environmental noise exposure (65–100 dB SPL) has emerged as a public health issue, humans typically experience such noises in industrial or occupational settings, as well as from traffic [14,15]. Furthermore, studies indicate that although moderate noise does not alter the auditory sensitivity of rats [16], prolonged exposure to environmental noise is associated with various neuropsychiatric diseases, including anxiety, depression, PD, and Alzheimer’s disease [17–22]. Recent clinical investigations have associated road traffic noise (>65 dB SPL) with an increased risk of exacerbating PD, while for every dB that noise levels fell, daily PD-hospital admissions and PD-ambulatory visits significantly decreased [22]. However, the effects of nontraumatic, high-decibel environmental noise exposure on PD and its underlying mechanisms remain unclear.

The main pathological feature of PD is the selective loss of dopaminergic (DA) neurons in the substantia nigra compacta (SNc) [23,24]. When PD patients develop motor deficits, usually more than 60% of SNc^DA^ neurons have already died [25]. It is generally believed that SNc^DA^ neurons are mainly related to physiological motor function [26,27]. However, recent studies have shown that SNc^DA^ neurons can participate in the transmission and processing of sensory information such as vision or hearing [28–30]. Besides, a study also revealed that long-term psychological stress can activate the projection from central amygdala (CeA) nucleus to SNc^DA^, leading to further loss of SNc^DA^ neurons in PD animal model [31]. Thus, we aimed to clarify whether the auditory-related brain regions are involved in PD with environmental noise exposure and whether chronic environmental noise exposure further leads to the loss of SNc^DA^ neurons.

To investigate the effect of environmental noise on PD severity, we first used low-dose 6-hydroxydopamine (6-OHDA) to establish an early-stage PD mouse model. Previous study has indicated that bilateral injection of low-dose 6-OHDA into dorsal striatum (STR) resulted in partial damage to DA neurons, but did not lead to changes in motor ability [32]. Subsequently, acute noise exposure (85–100 dB, 1-hour for 1 day) and chronic noise exposure models (85–100 dB, 1-hour per day for 7 days) were developed. The noise exposure protocol was based on a survey that showed noise levels are not less than 85 dB SPL in some industrial trades and environments [33,34]. White noise was chosen for this study due to its inclusion of all audible spectrum frequencies and its common use in laboratory experiments simulating environmental noise, such as assessing the effects of sleep and stress hormones on rats, as well as the impacts of insulin resistance and immunity in mice [35–37]. Locomotion test, rotarod test, and gait test were employed to assess the motor ability of mice. Additionally, immunofluorescence staining with anti-tyrosine hydroxylase (TH) was used to detect the loss of DA neurons in the SNc.

In order to understand the potential neural circuit mechanism underlying environmental noise-induced PD motor deficits, virus tracing strategy and fiber photometry were used to clarify the structural and functional connectivity of the projections from the inferior colliculus (IC) to SNc^DA^ neurons. Optogenetics and chemogenetic tools were also used to artificially manipulate IC-SNc^DA^ circuit to simulate or reverse environmental noise-induced motor deficits. Subsequently, RNA sequencing (RNA-Seq) was employed to explore the SNc molecular changes induced by environmental noise exposure. Overexpression of the downregulated gene at IC-SNc^DA^ circuit was utilized to evaluate the therapeutic effect of vesicular monoamine transporter 2 (VMAT2) activation on environmental noise exposure-induced motor deficits in 6-OHDA mice. Our study thus uncovered a neural circuit mechanism for SNc^DA^ neurons to receive thalamic auditory signals of environmental noise in modulating motor performance and neuronal susceptibility during the progression of early-stage PD.

## Results

### Acute noise exposure induces reversible motor deficits in 6-OHDA mice

**Fig 1:**
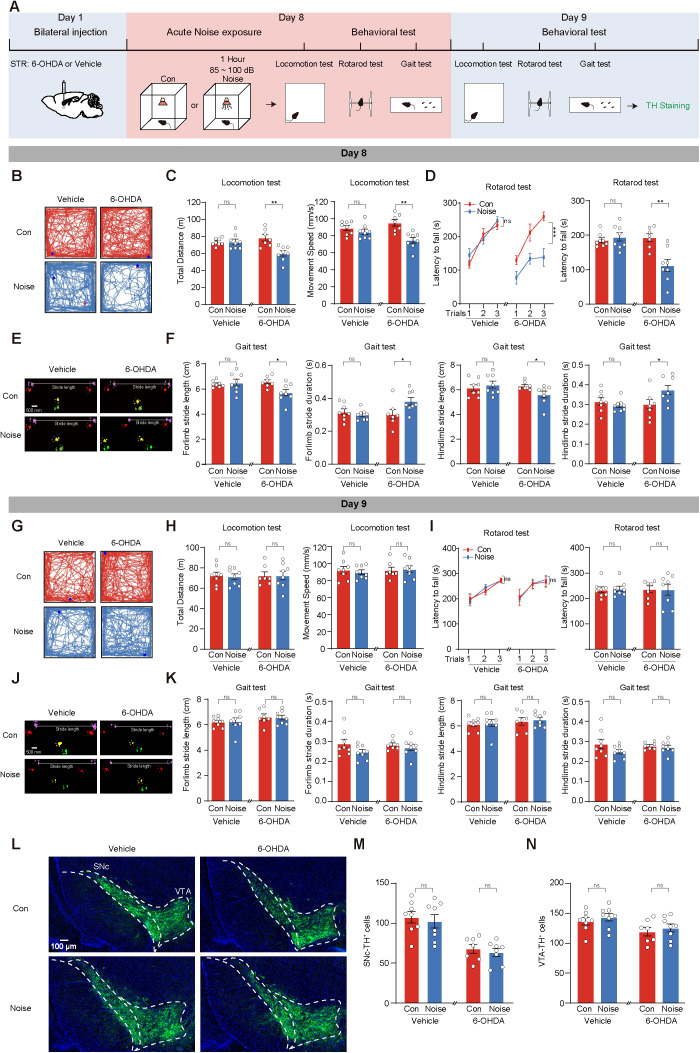
Acute noise exposure induces reversible motor deficits in 6-hydroxydopamine (6-OHDA) mice. **(A)** Experimental paradigm for establishing acute noise exposure model and measuring movement behaviors at day 8 and day 9 using locomotion test, rotarod test, and gait test. Vehicle+Control (Con) group (*n* = 8), Vehicle+Noise group (*n* = 8), 6-OHDA+CON group (*n* = 7), 6-OHDA+Noise group (*n* = 8). **(B–F)** Representative traces and statistics of mice in locomotion test (B, C), rotarod test (D), and gait test (E, F) on day 8. **(G–K)** Traces and statistics representing mice performance in locomotion test (G, H), rotarod test (I), and gait test (J, K) on day 9. **(L)** Representative images of tyrosine hydroxylase (TH) immunofluorescence in SNc and ventral tegmental area (VTA). **(M, N)** Statistics results of TH^+^ cells in SNc (M) and VTA (N). Data are presented as the mean ± SEM. *P < 0.05, **P < 0.01, ***P < 0.001, and ns for no significance. Unpaired *t* test and two-way ANOVA were used in panel. The data underlying this figure can be found in S1 Data.

To investigate the impact of acute noise exposure on motor function in early-stage PD, we used a subacute low-dose 6-OHDA model in C57BL/6 mice. Mice received bilateral injections of 6-OHDA (0.75 μl, 3 mg/ml) into the STR to induce the early-stage PD before the onset of motor symptoms, while control mice received vehicle injections on day 1. Seven days later, mice were exposed to white noise (85–100 dB) for 1 h in a soundproof chamber. Motor function was assessed immediately after noise exposure on day 8 and again 24 h later on day 9 using locomotion, rotarod, and gait tests. Following behavioral testing, mice were euthanized for TH staining in the SNc, with the ventral tegmental area (VTA) as a control (Fig 1A).

After acute noise exposure, the 6-OHDA+Noise mice showed significantly reduced total distance traveled and movement speed in the locomotion test compared to Vehicle+CON, Vehicle+Noise, or 6-OHDA+CON mice (Fig 1B). They also exhibited shorter latency to fall in the rotarod test (Fig 1D) and poorer performance in stride length and duration during the gait test (Fig 1E and 1F), indicating acute noise exposure induced motor deficits in the 6-OHDA model.

One day post-exposure, there were no significant differences in locomotion, rotarod performance, or gait parameters between 6-OHDA+Noise and control groups (Fig 1G–1K). Acute noise exposure did not affect Vehicle mice in any measured parameters (Fig 1B–1K). Immunostaining for TH^+^ neurons in the SNc and VTA revealed no differences between 6-OHDA+CON and 6-OHDA+Noise mice (Fig 1L–1N), suggesting that acute noise exposure reversibly impaired motor function in the early-stage PD model without exacerbating DA neuron loss.

In addition, we established an MPTP mouse model and administered an acute noise exposure. The results were similar to those of the 6-OHDA model, where MPTP mice also developed apparent motor deficits following the acute noise exposure (S1 Fig).

In summary, acute noise exposure transiently impaired motor function in early-stage PD mice, highlighting potential environmental influences on PD progression.

### Chronic noise exposure leads to irreversible motor deficits and SNc dopaminergic neuronal loss in 6-OHDA mice

**Fig 2:**
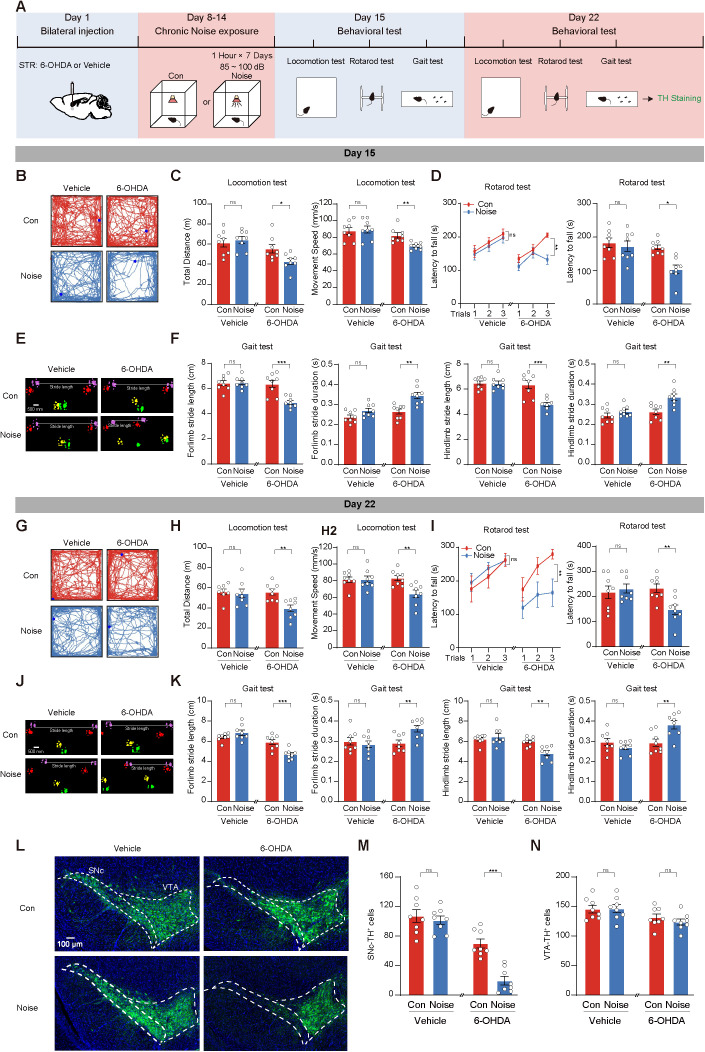
Chronic noise exposure leads to irreversible motor deficits and substantia nigra compacta (SNc) dopaminergic neuronal loss in 6-hydroxydopamine mice. **(A)** The timeline of experimental scheme and diagram for chronic noise exposure model. *n* = 8 mice for each group. **(B–F)** Representative traces and statistics of mice in locomotion test (B, C), rotarod test (D), and gait test (E, F) on day 15. **(G–K)** Traces and statistics representing mice performance in locomotion test (G, H), rotarod test (I), and gait test (J, K) on day 22. **(L)** Representative images of tyrosine hydroxylase (TH) immunofluorescence in SNc and ventral tegmental area (VTA). **(M, N)** Statistics results of TH^+^ cells in SNc (M) and VTA (N). Data are showed as the mean ± SEM. *P < 0.05, **P < 0.01, ***P < 0.001, and ns for no significance. Unpaired *t* test, Mann–Whitney *U* test, and two-way ANOVA were used in this figure. The data underlying this figure can be found in S2 Data.

To further explore the impact of chronic noise exposure on early-stage PD, we developed a model of chronic noise exposure in 6-OHDA mice. After injecting 6-OHDA into the STR, mice underwent chronic exposure to white noise (85–100 dB) for 7 days (1 h per day). Subsequently, we evaluated the motor function of all mice at 1 day and 7 days after the final noise exposure session. Mice were then euthanized to assess the number of TH^+^ neurons in both the SNc and VTA regions (Fig 2A).

One day after chronic noise exposure, the locomotion test revealed a significant decrease in total distance traveled and movement speed in 6-OHDA+Noise mice compared to control groups (Fig 2B and 2C). Additionally, 6-OHDA+Noise mice exhibited reduced latency to fall on the rotarod (Fig 2D) and impaired performance in stride length and duration during the gait test (Fig 2E and 2F). However, chronic noise exposure alone did not affect the motor function of vehicle-injected mice (Fig 2B–2F).

One week later, 6-OHDA+Noise mice continued to show significant decreases in locomotion, rotarod performance, and gait parameters compared to 6-OHDA+CON mice (Fig 2G–2K). No significant differences were observed between Vehicle+CON and Vehicle+Noise mice (Fig 2G–2K). Importantly, we observed a significant reduction in the number of SNc TH^+^ neurons in 6-OHDA+Noise mice compared to 6-OHDA+CON mice, while there was no difference in the number of VTA TH^+^ neurons (Fig 2L–2N).

Furthermore, we conducted validation studies using the MPTP model, which demonstrated that MPTP mice exhibited irreversible motor deficits following 7 days of chronic noise exposure (S2 Fig).

These findings suggest that chronic noise exposure induces irreversible motor deficits and loss of SNc TH^+^ neurons in the early-stage PD mouse model.

Moreover, numerous studies have shown that music can alleviate PD symptoms [38,39]. To investigate this further, we replaced noise exposure with the similar high-decibel music (85–100 dB). These results showed that PD mice exposed to acute or chronic high-decibel music exposure also displayed significant motor deficits compared to the control group (S3 and S4 Figs), suggesting that the noise exposure-induced motor deficits were predominantly associated with high-decibel sound, rather than the types of sound.

### IC neurons respond to noise stimulation and project to SNc dopaminergic neurons

**Fig 3:**
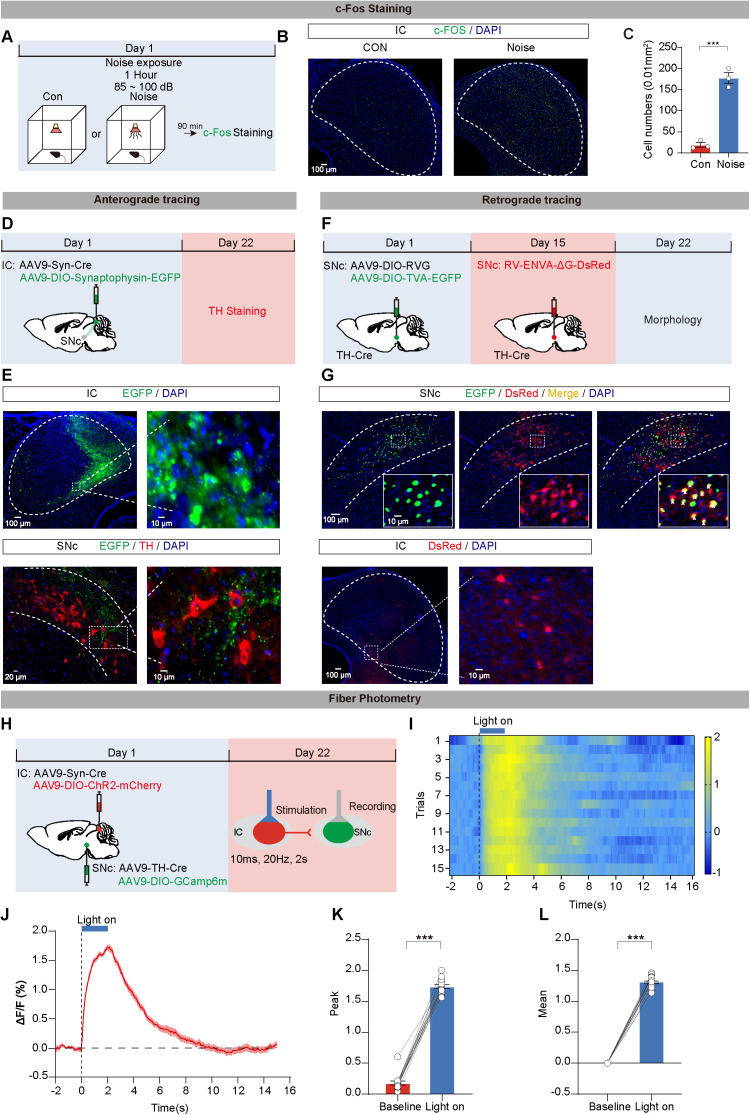
Inferior colliculus (IC) neurons respond to noise stimulation and project to substantia nigra compacta (SNc) dopaminergic neurons. **(A)** Experimental diagram for c-FOS staining activated by noise in IC. **(B)** Representative images of c-FOS immunofluorescence in IC of CON and Noise mice. **(C)** Statistics results of c-FOS^+^ cells in CON and Noise mice. *n* = 3 mice for each group. **(D)** Virus strategy for tracing the circuit from IC neurons to SNc^DA^ neurons. **(E)** Fluorescence images showing EGFP^+^ cells (green) in IC (upper panel) and EGFP^+^ projections (green) and TH^+^ cells (red) in SNc (lower panel). **(F)** Virus strategy for *trans-*monosynaptic retrograde tracing the circuit from IC neurons to SNc^DA^ neurons. **(G)** Fluorescence images of starter cells (yellow) in SNc, which co-infected by AAV9-DIO-RVG, AAV9-DIO-TVA-EGFP (green), and RV-ENVA-ΔG-DsRed (red) in TH-Cre transgenic mice (upper panel) and DsRed-labeled neurons in the IC traced from SNc^DA^ neurons (lower panel). **(H)** Schematic of virus injection, stimulation, and fluorescence recordings. **(I)** Heatmaps of Ca^2+^ transients evoked by blue light stimulation (473 nm, 20 Hz, 10 ms pulse width, 2 s duration, 18 s interval) of ChR2-expressing cells in IC. **(J)** Average plot of Ca^2+^ responses in mice. **(K, L)** Statistics results of peak (K) and mean (L) ΔF/F of fluorescence signals in the pre-light session (Baseline group) and light stimulation session (Light on group). Data are displayed as the mean ± SEM. *P < 0.05, **P < 0.01, ***P < 0.001, and ns for no significance. Unpaired *t* test and Wilcoxon signed-rank test were used in this figure. The data underlying this figure can be found in S3 Data.

The IC serves as a primary hub for integrating auditory information from external environment. In our study, we focused on the IC to investigate its response to noise stimulation and its connections to SNc^DA^ neurons. Initially, we assessed the activation of IC neurons following noise exposure using immunofluorescence staining (Fig 3A). Our findings indicated a significant increase in c-FOS^+^ cell numbers in the IC of noise-exposed mice compared to controls (Fig 3B and 3C). To further validate that environmental noise drives SNc^DA^ neuronal activity, we performed in vivo electrophysiological recordings. The results demonstrated that noise exposure significantly increased the firing frequency of putative SNc^DA^ neurons, whereas inhibition of IC neurons further reduced their firing frequency compared to the pre-stimulation baseline. These findings indicate that environmental noise modulates SNc^DA^ neuronal activity through a monosynaptic IC-SNc^DA^ circuit (S5 Fig).

Subsequently, using anterograde and *trans-*monosynaptic retrograde viral tracing methods, we morphologically verified that IC neurons project monosynaptically to SNc dopaminergic neurons. For anterograde tracing, AAV9-hSyn-Cre and AAV9-DIO-Synaptophysin-EGFP were injected into the IC to label axon terminals of IC projection neurons with synaptophysin fused to EGFP (Figs 3D and S6). EGFP expression was localized within the IC neuronal cell bodies, and immunofluorescence confirmed dense green EGFP-labeled terminals surrounding TH^+^ neurons marked in red in the SNc region (Figs 3E and S6). For retrograde tracing, the IC-SNc^DA^ circuit was further verified through cell-type-specific *trans-*monosynaptic retrograde tracing from SNc^DA^ neurons. Cre-dependent helper viruses (AAV9-DIO-TVA-EGFP and AAV9-DIO-RVG) were injected into the SNc of TH-Cre transgenic mice, followed by injection of RV-ENVA-ΔG-DsRed after 14 days (Figs 3F and S7). Histological analysis 1 week later revealed EGFP^+^/DsRed^+^ starter cells restricted to the SNc injection site, with numerous DsRed-expressing neurons observed in the IC (Figs 3G and S6), stereological quantification was employed to assess the number of IC-SNc^DA^ neuronal somata and synapses (S8 Fig). These results indicated direct projections from IC neurons to SNc^DA^ neurons.

To assess functional connectivity, simultaneous fiber photometry recordings and optogenetic stimulation were used (Fig 3H). Optogenetic activation of IC ChR2-expressed neurons under blue light stimulation resulted in a significant increase in calcium fluorescence signals in SNc^DA^ neurons compared to baseline, suggesting that IC neuron excitation triggered SNc^DA^ neuron activation (Fig 3I–3L). Moreover, we utilized in vivo electrophysiology to record the single-unit activity of putative SNc^DA^ neurons during light activation, suggesting that activation of IC neurons can evoke firing in SNc^DA^ neurons, demonstrating the functional connectivity of IC-SNc^DA^ circuit (S9 Fig). Furthermore, we specifically expressed a glutamate sensor in SNc^DA^ neurons and injected AAV9-Syn-Cre combined with AAV9-DIO-ChR2-mCherry into the IC. The results suggested that light stimulation elicited a significant increase in glutamate release in the SNc, while NBQX blocked this enhancement (S10 Fig).

In summary, these findings highlight the role of the IC-SNc circuit in responding to noise stimulation and projecting to SNc^DA^ neurons, elucidating potential mechanisms underlying noise-induced motor deficits and neuronal loss in early-stage PD.

### Short-term modulation of IC-SNc circuit affects motor deficits induced by acute noise exposure in 6-OHDA mice

**Fig 4:**
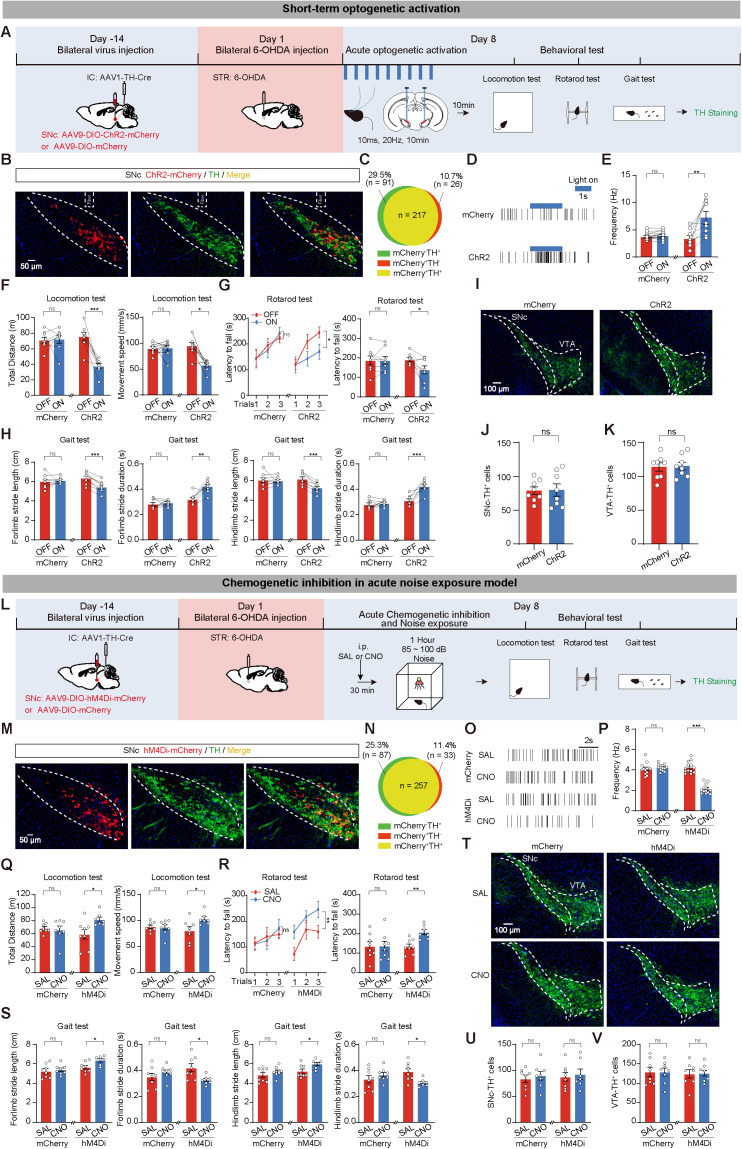
Short-term modulation of inferior colliculus (IC)-substantia nigra compacta (SNc) circuit affects motor deficits induced by acute noise exposure in 6-hydroxydopamine mice. **(A)** Experimental paradigm for short-term optogenetic activation of IC-SNc^DA^ circuit. **(B)** Representative images of SNc injection sites. Red channel: The viral expression of AAV1-TH-Cre and AAV9-DIO-ChR2-mCherry (ChR2) or AAV9-DIO-mCherry (mCherry) in SNc. Green channel: immunofluorescence staining of anti-tyrosine hydroxylase (TH). **(C)** The quantitative analysis of the Venn diagram reveals the extent of co-expression between mCherry and TH in the SNc. **(D, E)** Representative raster plots and statistical results depicting the firing rates of spontaneous spikes in putative SNc^DA^ neurons. **(F–H)** Statistics results of locomotion test (F), rotarod test (G), and gait test (H). *n* = 8 mice for each group. **(I)** Representative images of anti-TH immunofluorescence in SNc and VTA. **(J, K)** Statistics results of TH^+^ cells in SNc (J) and ventral tegmental area (VTA) (K). **(L)** Schematic of viral strategy and chemogenetic inhibition of IC-SNc^DA^ circuit in acute noise exposure model. **(M)** Representative images of viral expression of mCherry (red) in SNc TH^+^ cells (green). **(N)** The quantitative analysis of Venn diagram shows the co-expression level of mCherry with TH in SNc. **(O, P)** Raster graphs and firing rate of hM4Di and mCherry mice after saline (SAL) or CNO injection. **(Q–S)** Statistics results of locomotion test (Q), rotarod test (R), and gait test (S). mCherry+SAL group (*n* = 8), mCherry+CNO group (*n* = 8), hM4Di+SAL group (*n* = 8), hM4Di+CNO group (*n* = 7). **(T)** Representative images of TH immunofluorescence in SNc and VTA. **(U, V)** Statistics results of TH^+^ cells in SNc (U) and VTA (V). Data are exhibited as the mean ± SEM. *P < 0.05, **P < 0.01, ***P < 0.001, and ns for no significance. Paired *t* tests, Wilcoxon signed-rank tests, unpaired *t* tests, Mann–Whitney *U* test, and two-way ANOVA were used in this figure. The data underlying this figure can be found in S4 Data.

Based on the preceding findings, we aimed to ascertain whether short-term activation of the IC-SNc^DA^ circuit could replicate the effects of acute noise exposure on the 6-OHDA model. All mice were bilaterally injected with AAV1-TH-Cre into the IC, facilitating anterograde transport from neuronal cell bodies to axon terminals and subsequently through a single synapse to connected neurons. Additionally, AAV9-DIO-ChR2-mCherry (ChR2 group) was infused into the SNc, while AAV9-DIO-mCherry served as the control (mCherry group). Following two weeks of viral expression, all mice received 6-OHDA injections. On day 8, mice underwent 10 min of blue light stimulation to activate the IC-SNc^DA^ circuit, followed by a series of behavioral tests. Finally, mice were euthanized to detect TH^+^ cells (Fig 4A). Immunofluorescence analysis confirmed that ChR2-mCherry expression was limited to DA neurons in the SNc region (Figs 4B, 4C, and S11 Fig). Blue light stimulation significantly activated putative SNc^DA^ neurons as evidenced by in vivo electrophysiology recordings (Fig 4D and 4E). The ChR2 group exhibited significant motor deficits in locomotion, rotarod, and gait tests after blue light stimulation (Fig 4F–4H). Furthermore, immunofluorescence staining revealed that acute activation of the IC-SNc^DA^ circuit did not lead to increased loss of TH^+^ neurons in the SNc (Fig 4I–4K). Moreover, we employed different viral strategies to either activate the IC-SNc circuit. The results were consistent with optogenetics, short-term activation of the IC-SNc circuit leads to motor deficits in 6-OHDA mice without exacerbating SNc^DA^ neurons loss (S12 Fig).

To evaluate whether short-term inhibition of the IC-SNc^DA^ circuit reverses the effects of acute noise exposure in the 6-OHDA model, we bilaterally injected AAV1-TH-Cre into the IC and AAV9-DIO-hM4Di-mCherry (hM4Di group) into the SNc of WT mice, while AAV9-DIO-mCherry (mCherry group) served as the control. Following 2 weeks of viral expression, all mice received 6-OHDA injections. Thirty minutes after saline (SAL) or CNO injection, mice were subjected to acute noise exposure on day 8. After a series of behavioral tests, we euthanized the mice to detect TH^+^ neurons (Fig 4L). Immunofluorescence results suggested that hM4Di-mCherry expression was confined to DA neurons in the SNc region (Fig 4M and 4N). CNO injection significantly suppressed the activity of putative SNc^DA^ neurons (Fig 4O and 4P). Compared to the hM4Di+SAL group, the hM4Di+CNO group exhibited increased total distance and movement speed in the locomotion test, increased latency to fall in the rotarod test, increased stride length, and decreased stride duration in the gait test (Fig 4Q–4S). Similarly, immunofluorescence staining indicated that there was no significant difference in the number of SNc and VTA TH^+^ neurons (Fig 4T–4V).

Together, these findings suggest that short-term bilateral modulation of the IC-SNc^DA^ circuit can mimic or reverse acute noise exposure-induced motor deficits in the 6-OHDA model.

### Long-term bidirectional modulation of IC-SNc circuit mimics or reverses chronic noise exposure-induced motor deficits and SNc dopaminergic neuronal loss in 6-OHDA mice

**Fig 5:**
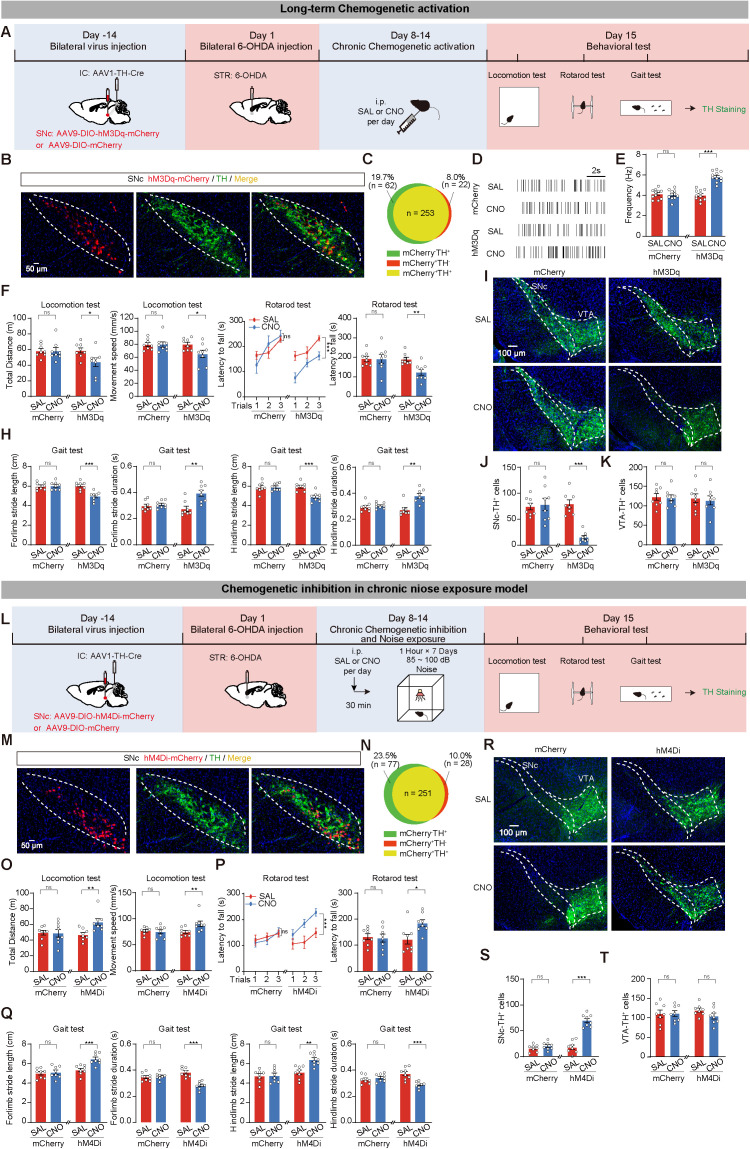
Long-term bidirectional modulation of inferior colliculus (IC)-substantia nigra compacta (SNc) circuit mimics or reverses chronic noise exposure-induced motor deficits and SNc dopaminergic neuronal loss in 6-hydroxydopamine mice. **(A)** Experimental paradigm for long-term chemogenetic activation of IC-SNc^DA^ circuit. **(B)** Representative images of SNc injection sites. Red channel: The viral expression of AAV1-TH-Cre and AAV9-DIO-hM3Dq-mCherry (hM3Dq) in SNc. Green channel: immunofluorescence staining of tyrosine hydroxylase (TH). **(C)** Quantitative analysis of the co-expression between mCherry and TH in the SNc. **(D, E)** Representative raster plots (D) and statistical results (E) depicting the firing rates of spontaneous spikes in putative SNc^DA^ neurons. **(F–H)** Statistics results of locomotion test (F), rotarod test (G), and gait test (H). *n* = 8 mice for each group. **(I)** Representative images of TH immunofluorescence in SNc and ventral tegmental area (VTA). **(J, K)** Statistics results of TH^+^ cells in SNc (J) and VTA (K). **(L)** Schematic of viral strategy and chemogenetic inhibition of IC-SNc^DA^ circuit in chronic noise exposure model. **(M)** Representative images of viral expression of mCherry (red) in SNc TH^+^ cells (green). **(N)** The quantitative analysis of Venn diagram shows the co-expression level of mCherry with TH in SNc. **(O–Q)** Statistics results of locomotion test (O), rotarod test (P), and gait test (Q). *n* = 8 mice for each group. **(R–T)** Representative images (R) and statistics results of immunofluorescence with anti-TH in SNc (S) and VTA (T). Data are displayed as the mean ± SEM. *P < 0.05, **P < 0.01, ***P < 0.001, and ns for no significance. Unpaired *t* test, Mann–Whitney *U* test, and two-way ANOVA were used in this figure. The data underlying this figure can be found in S5 Data.

Furthermore, we tested the effects of long-term activation or inhibition of the IC-SNc circuit in the chronic noise exposure 6-OHDA model. We bilaterally injected AAV1-TH-Cre into the IC of WT mice, followed by bilateral injection of AAV9-DIO-hM3Dq-mCherry (hM3Dq group) into the SNc, with AAV9-DIO-mCherry as the control (mCherry group). After 2 weeks, all mice received 6-OHDA injections. From the 8th day, the mice received SAL or CNO injections daily for 7 consecutive days. On the 15th day, a series of behavioral tests were administered. Finally, the mice were euthanized to detect TH^+^ neurons (Fig 5A). The expression of hM3Dq in SNc^DA^ neurons was confirmed through co-staining with TH (Fig 5B and 5C). The activity of SNc^DA^ neurons expressing hM3Dq-mCherry was significantly activated by CNO (Fig 5D and 5E). Mice that received CNO administration with hM3Dq expression exhibited decreased total distance and movement speed in the locomotion test, decreased latency to fall in the rotarod test, decreased stride length, and increased stride duration in the gait test (Fig 5F–5H). Moreover, brain tissue immunofluorescence staining revealed that the loss of TH^+^ cells was increased in the SNc of hM3Dq+CNO mice but not in the VTA (Fig 5I–5K).

To determine the effects of long-term inhibition of the IC-SNc circuit, we injected AAV9-DIO-hM4Di-mCherry (hM4Di group) or AAV9-DIO-mCherry (mCherry group) as a control into the SNc, while AAV1-TH-Cre was injected into the IC. After 2 weeks of viral expression, we injected 6-OHDA into the STR of mice. From day 8 to day 14, all mice were subjected to 1-hour noise exposure daily and received either SAL or CNO injection half an hour before the noise exposure. On the 15th day, the same series of behavioral tests were carried out. Finally, the mice were euthanized to detect TH^+^ neurons (Fig 5L). The expression of hM4Di was confirmed to be co-expressed with TH in SNc^DA^ neurons (Fig 5M and 5N). Behavioral tests showed that hM4Di+CNO mice had increased total distance and faster movement speed in the locomotion test, more latency time to fall off the rotarod in the rotarod test, and better performance in the gait test compared to control groups (Fig 5O–5Q). Immunofluorescence staining indicated no significant difference in the number of TH^+^ neurons in the VTA among four groups. However, the SNc showed a greater number of TH^+^ neurons in the hM4Di+CNO group, indicating that inhibition of the IC-SNc^DA^ circuit rescued SNc^DA^ neuron loss (Fig 5R–5T). Furthermore, we employed distinct viral-mediated approaches to selectively inhibit the IC-SNc circuit. The results also showed that prolonged circuit inhibition not only rescued chronic noise exposure-induced motor deficits in 6-OHDA mice but also exerted neuroprotective effects on SNc^DA^ neurons (S13 Fig).

To further validate the critical role of the IC-SNc circuit in noise exposure, we established a PD model by bilateral injecting of AAV9-Syn-A53T-α-synuclein into the SNc, with control group receiving AAV9-Syn. The results showed that compared with the A53T+CNO group, the A53T+SAL group exhibited significant motor deficits, while the number of SNc^DA^ neurons was significantly reduced (S14 Fig).

Taken together, these results suggest that long-term activation or inhibition of the IC-SNc^DA^ circuit can mimic or reverse the motor deficits and susceptibility to 6-OHDA induced by chronic noise exposure. Moreover, the injection of AAV9 alone did not lead to a reduction in SNc^DA^ neurons (S15 Fig).

### Noise exposure and IC-SNc circuit activation reduce VMAT2 expression in SNc

**Fig 6:**
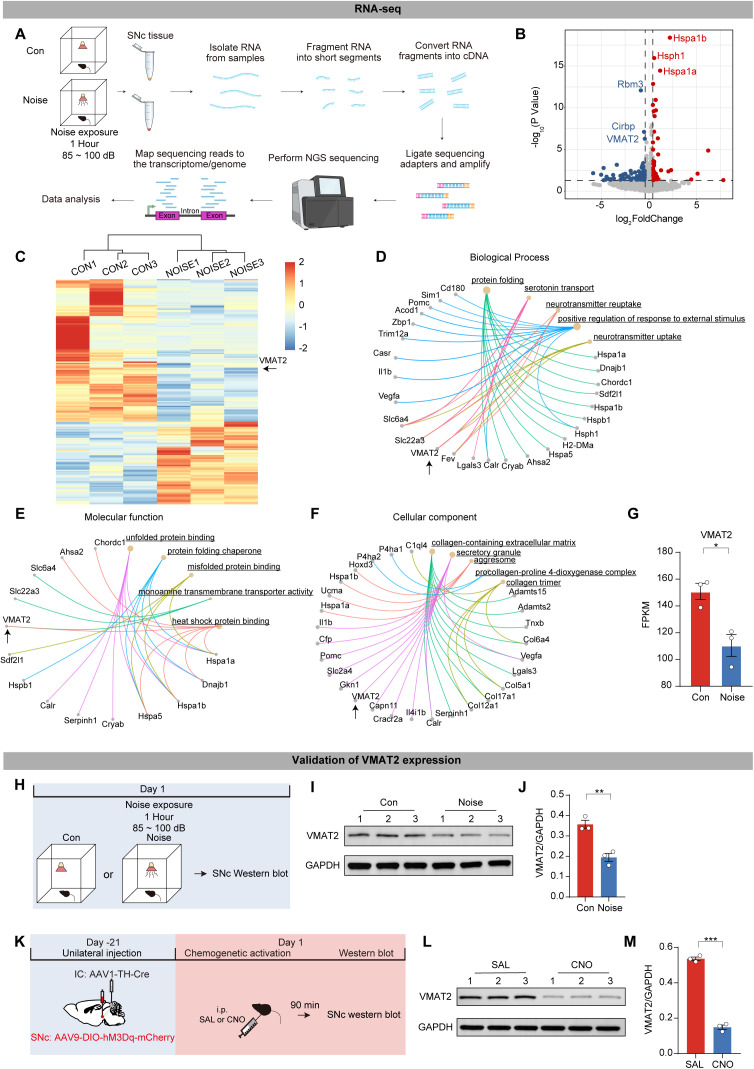
Noise exposure or activation of inferior colliculus (IC)-substantia nigra compacta (SNc) circuit reduce vesicular monoamine transporter 2 (VMAT2) expression in SNc. **(A)** Workflow of RNA sequencing (RNA-Seq) for the SNc tissue in Con and Noise mice. **(B)** Volcano plot showing differentially expressed genes (DEGs) in SNc of CON and noise mice. *n* = 3 mice for each group. **(C)** Heatmap of DEGs determined by RNA-Seq of CON and noise mice. **(D–F)** Gene Ontology enrichment analysis of the DEGs. **(G)** Quantitative analysis of vesicular monoamine transporter 2 (VMAT2) expression in RNA-Seq. **(H)** Experimental diagram for validation of VMAT2 in noise exposure model. **(I)** Representative western blotting for VMAT2 expression in noise exposure model. **(J)** Quantitative analysis of VMAT2 expression in noise exposure model. *n* = 3 mice for each group. **(K)** Experimental diagram for validation of VMAT2 in IC-SNc^DA^ circuit activation by chemogenetic tool. **(L)** Representative western blotting for VMAT2 expression in chemogenetic activation experiment. **(M)** Quantitative analysis of VMAT2 expression in chemogenetic activation experiment. *n* = 3 mice for each group. Data are showed as the mean ± SEM. *P < 0.05, **P < 0.01, ***P < 0.001, and ns for no significance. Unpaired *t* test was used in this figure. The data underlying this figure can be found in S6 Data.

To elucidate the molecular mechanisms behind the effects of noise exposure on early-stage PD, we employed RNA-Seq to identify differentially expressed genes (DEGs) in the SNc due to noise exposure (Fig 6A). The results revealed 69 significantly upregulated and 114 downregulated genes (Fig 6B). A heatmap and hierarchical clustering analysis displayed the expression patterns of these DEGs (Fig 6C). Gene Ontology (GO) enrichment analyses indicated that these 183 DEGs were involved in biological processes like protein folding, serotonin transport, and neurotransmitter reuptake, molecular functions such as unfolded protein binding, protein folding chaperone, misfolded protein binding, and monoamine transmembrane transporter activity, and cellular components related to collagen-containing extracellular matrix, secretory granules, and aggresomes (Fig 6D–6F). These findings suggest that changes in protein folding, vesicle secretion, and neurotransmitter transmission in the SNc may contribute to noise-induced motor deficits.

Previous experiments showed that acute noise exposure causes reversible motor deficits in PD mice, while chronic noise exposure results in permanent motor deficits and SNc^DA^ neuronal loss. We hypothesize that these effects are due to alterations in dopamine secretion and reuptake. VMAT2 is crucial in DA neurons, transporting dopamine from the cytoplasm into synaptic vesicles for storage and releasing it into the synaptic cleft for neurotransmitter reuptake. RNA-Seq data (FPKM) revealed significantly reduced VMAT2 expression in the SNc of noise-exposed mice (Fig 6G), identifying VMAT2 as a key gene of interest.

Western blotting analysis confirmed that VMAT2 protein levels in the SNc were significantly lower in the noise group compared to controls (Fig 6H–6J). To investigate the impact of IC-SNc^DA^ circuit activation on VMAT2 expression in the SNc, we injected AAV1-TH-Cre into the IC and AAV9-DIO-hM3Dq-mCherry into the SNc. After 3 weeks of recovery, SAL or CNO was intraperitoneally injected on day 1, and SNc tissue samples were collected 30 min postinjection (Fig 6K). We found that IC-SNc circuit activation significantly reduced VMAT2 protein expression in the SNc, even in the absence of noise exposure (Fig 6L and 6M).

These results suggest that both noise exposure and IC-SNc circuit activation decrease VMAT2 expression in the SNc, potentially contributing to motor deficits in the 6-OHDA model.

### VMAT2 overexpression in IC-innervated SNc dopaminergic neurons ameliorates noise exposure-induced 6-OHDA vulnerability

**Fig 7:**
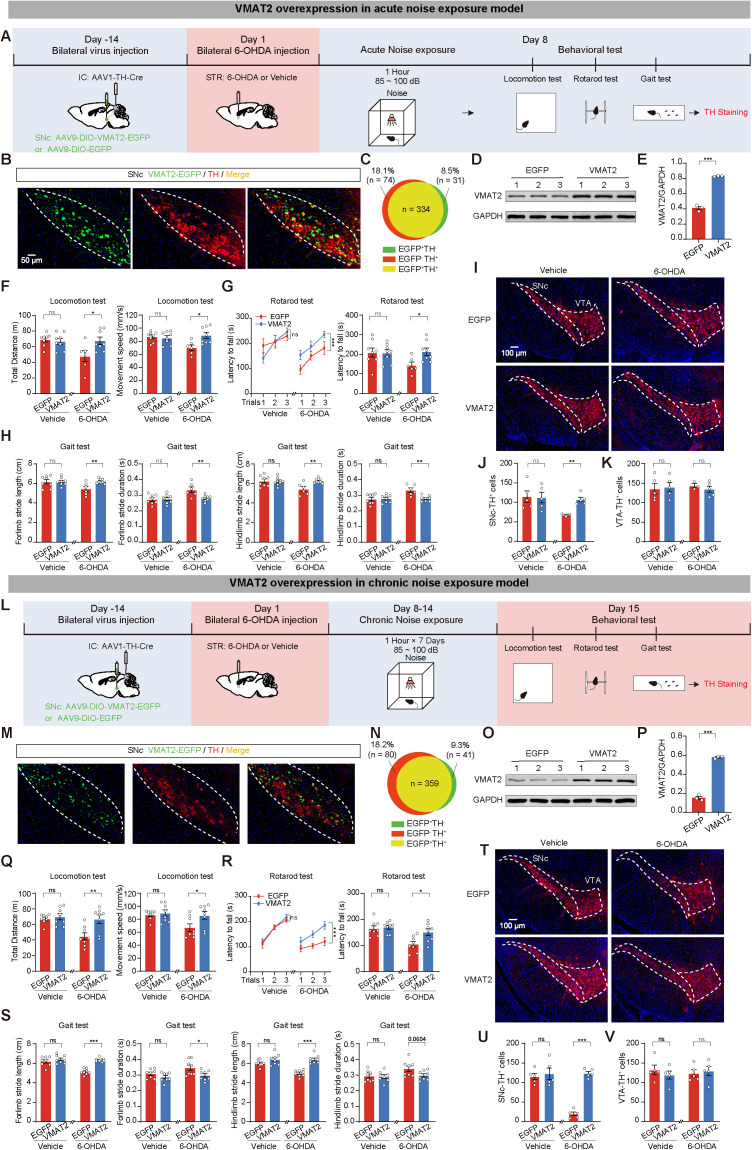
Vesicular monoamine transporter 2 (VMAT2) overexpression in inferior colliculus (IC)-innervated substantia nigra compacta (SNc) dopaminergic neurons ameliorates noise exposure-induced 6-hydroxydopamine (6-OHDA) vulnerability. **(A)** Experimental paradigm for VMAT2 overexpression at IC-SNc^DA^ circuit in acute noise exposure model. **(B)** Representative images of SNc injection sites. Green channel: Viral expression of AAV1-TH-Cre and AAV9-DIO-VMAT2-EGFP (VMAT2) in SNc. Red channel: immunofluorescence staining of tyrosine hydroxylase (TH). **(C)** Venn diagram reveals the quantitative analysis of co-expression between mCherry and TH in the SNc. **(D)** Representative western blotting for VMAT2 expression in four groups. **(E)** Quantitative analysis of VMAT2 expression in four groups. *n* = 3 mice for each group. **(F–H)** Statistics results of locomotion test (F), rotarod test (G), and gait test (H). Vehicle+EGFP group (*n* = 8), Vehicle+VMAT2 group (*n* = 8), 6-OHDA+EGFP group (*n* = 6), 6-OHDA+VMAT2 group (*n* = 8). **(I)** Representative images of anti-TH immunofluorescence in SNc and ventral tegmental area (VTA). **(J, K)** Statistics results of TH^+^ cells in SNc (J) and VTA (K). **(L)** Experimental paradigm for overexpression of VMAT2 of IC-SNc^DA^ circuit in chronic noise exposure model. **(M)** Representative images of SNc injection sites. Green channel: The viral expression of AAV1-TH-Cre and AAV9-DIO-VMAT2-EGFP (VMAT2) in SNc. Red channel: immunofluorescence staining of TH. **(N)** The quantitative analysis of Venn diagram shows the co-expression level of mCherry with TH in SNc. **(O)** Representative western blotting for VMAT2 expression in four groups. **(P)** Quantitative analysis of VMAT2 expression in four groups. *n* = 3 mice for each group. **(Q–S)** Statistics results of locomotion test (Q), rotarod test (R), and gait test (S). *n* = 8 mice for each group. **(T)** Representative images of TH immunofluorescence in SNc and VTA. **(U, V)** Statistics results of TH^+^ cells in SNc (U) and VTA (V). Data are presented as the mean ± SEM. *P < 0.05, **P < 0.01, ***P < 0.001, and ns for no significance. Unpaired *t* test, Mann–Whitney *U* test, and two-way ANOVA were used in this figure. The data underlying this figure can be found in S7 Data.

We injected AAV1-TH-Cre into the IC and either AAV9-DIO-VMAT2-EGFP (VMAT2 group) or AAV9-DIO-EGFP (EGFP group) into the SNc. This facilitated the overexpression of VMAT2 in IC-innervated SNc^DA^ neurons. After 2 weeks of viral expression, we injected 6-OHDA or vehicle into the STR. On day 8, mice underwent 1 h of noise exposure, followed by a series of behavioral tests. Finally, we examined the number of TH^+^ cells in the SNc and VTA (Fig 7A). Histological analysis showed that VMAT2-EGFP expression was confined to SNc^DA^ neurons (Fig 7B and 7C). Immunoblotting validated the significant increase of VMAT2 expression in the VMAT2 group compared to the EGFP group (Fig 7D and 7E). Behavioral tests indicated that the 6-OHDA+VMAT2 group exhibited greater total distance and faster movement speed in the locomotion test, longer latency to fall in the rotarod test, and better performance in the gait test compared to the 6-OHDA+EGFP group (Fig 7F–7H). Additionally, immunofluorescence staining showed that VMAT2 overexpression partially rescued the loss of SNc TH^+^ neurons in the 6-OHDA model (Fig 7I–7K).

Furthermore, the effects of VMAT2 overexpression on chronic noise exposure in 6-OHDA model were measured. AAV9-DIO-VMAT2-EGFP (VMAT2 group) or AAV9-DIO-EGFP (EGFP group) was infused into the SNc, and AAV1-TH-Cre was bilaterally injected into the IC. After 2 weeks, we injected 6-OHDA or vehicle into the STR. From day 8 to day 14, mice were subjected to 1 h of noise exposure daily. On day 15, we performed the same behavioral tests and then euthanized the mice to detect TH^+^ neurons (Fig 7L). Immunofluorescence confirmed that VMAT2-EGFP expression was primarily restricted to TH^+^ cells in the SNc (Fig 7M and 7N). Western blot analysis confirmed VMAT2 overexpression in the VMAT2 group (Fig 7O and 7P). Compared to control groups, the 6-OHDA+VMAT2 group exhibited increased total distance and movement speed in the locomotion test, longer latency to fall in the rotarod test, increased stride length, and decreased stride duration in the gait test (Fig 7Q–7S). Immunofluorescence staining revealed a greater number of TH^+^ neurons in the SNc of the 6-OHDA+VMAT2 group (Fig 7T–7V).

These data collectively indicate that VMAT2 overexpression in the IC-innervated SNc^DA^ neurons effectively rescues noise exposure-induced 6-OHDA vulnerability.

## Discussion

Although numerous studies have reported that environmental factors play a key role in the development of PD, it remains unclear how environmental noise exposure affects PD severity [6,40,41]. In this study, we established both acute and chronic high-decibel noise exposure models to explore the relationship between environmental noise exposure and PD. We found that acute noise exposure led to reversible motor deficits in early-stage PD mouse model, without significantly altering the loss of SNc^DA^ neurons. However, chronic noise exposure made mice more susceptible to the effects of 6-OHDA insult, resulting in irreversible motor deficits and a reduction in SNc^DA^ neurons. Subsequently, we combined immediate early gene c-FOS expression, anterograde and retrograde viral tracing methods, calcium signaling recording, and behavioral tests to identify the IC as a key upstream nucleus for noise signals information processing to the SNc^DA^ neurons. Additionally, manipulating the IC-SNc^DA^ circuit using optogenetics or chemogenetics could bidirectionally modulate the impact of environmental noise on early-stage PD model. Mechanistically, this exacerbation effect was also improved by upregulating the expression of VMAT2. Overall, these findings uncovered a neural circuit mechanism for SNc^DA^ neurons to receive thalamic auditory signals of environmental noise in modulating motor behavior during the progression of early-stage PD.

The PD signs and symptoms are caused by high levels of DA neuronal death in the SNc and include tremors, bradykinesia, rigid muscles, speech and motor impairments, postural and balance disabilities, and difficulties in automatic movements [42]. In this study, we injected a low dose of 6-OHDA into the bilateral STR, causing partial death of SNc^DA^ neurons. Subsequently, we exposed mice to either acute or chronic noise to simulate the impact of environmental noise on PD patients. The results showed that compared to a single 6-OHDA challenge, the dual insult led to the onset of PD motor deficits. After 7 days of noise exposure, 6-OHDA mice exhibited more pronounced loss of DA neurons. In this “double-hit model”, all these findings support the critical role of environmental noise in PD severity.

Recent evidence suggested that SNc, as a part of the basal ganglia, plays a crucial role in processing and transmitting sensory information through its DA neurons [29,43]. Although the primary function of SNc is to receive projections from the motor-related nuclei for the modulation of motor control, recent studies have increasingly demonstrated its involvement in sensory information processing via direct projections from sensory nuclei [30,44]. For example, the projection from the superior colliculus to SNc^DA^ neurons transforms visual information into motor signals, triggering DA release in the STR and thereby facilitating appetitive behaviors in mice [28]. In the current study, we observed the remarkable activation of IC neurons by environmental noise exposure. Therefore, we propose an unexplored mechanism involving the role of IC-SNc^DA^ circuit in noise-induced motor deficits in the 6-OHDA mice. A recent study reported that activation of SNc^DA^ neurons can directly induce their own degeneration [45]. Therefore, we employed two distinct viral strategies to investigate the role of the IC-SNc circuit in noise-induced motor deficits. Both approaches demonstrated that activating or inhibiting this circuit bidirectionally modulates the effects of acute or chronic noise exposure on the 6-OHDA model.

To further investigate the molecular mechanism underlying the effect of noise exposure on early-stage PD model, we employed RNA-Seq analysis and gain-of-function genetic approach to demonstrate the crucial role of VMAT2 at the IC-SNc^DA^ circuit in this study. Previously published studies have suggested that VMAT2 is primarily expressed in the brain and plays a vital role in packaging and transporting monoamine neurotransmitters, such as DA, serotonin, and norepinephrine, into vesicles for synaptic release [46,47]. Reducing VMAT2 within DA neurons in mice diminishes the therapeutic efficacy of L-DOPA, indicating the significance of VMAT2 in DA transmission within the midbrain [48,49]. Additionally, it is well-documented that VMAT2 protects nigrostriatal DA neurons against 1-methyl-4-phenylpyridinium (MPP^+^) by sequestering it inside vesicles away from its mitochondrial site of neurotoxic action [50,51]. Another study has shown that transient inhibition of VMAT2 expression in mice with tetrabenazine, a reversible short-acting VMAT2 inhibitor, prior to 6-OHDA injection does not potentiate the toxicity of 6-OHDA to SNc^DA^ neurons [52]. Conversely, prolonged suppression of VMAT2 expression with reserpine pretreatment significantly enhances the toxicity of 6-OHDA to DA neurons [52]. These findings align with our discovery that acute noise exposure transiently reduces VMAT2 expression in SNc^DA^ neurons and leads to reversible motor deficits, whereas chronic noise exposure significantly increases 6-OHDA toxicity to SNc^DA^ neurons by continuously lowering VMAT2 expression and results in a reduction in SNc^DA^ neuron numbers and irreversible motor deficits in 6-OHDA mice. Furthermore, our study demonstrates that the noise exposure and IC-SNc^DA^ circuit activation induces a significant decrease of VMAT2 protein levels in the SNc. This reduction in VMAT2 protein may be linked to noise exposure, and previous studies have established a strong correlation between VMAT2 expression and stress-related factors [53,54]. Consequently, overexpression of VMAT2 at the IC-SNc^DA^ circuit markedly alleviates motor deficits induced by acute or chronic noise exposure, and also protects against chronic noise exposure-induced SNc^DA^ neurons loss.

In addition, the RNA-Seq results suggest the potential molecular mechanism. We performed differential genes enrichment analysis using GO to identify pathways responded to acute noise exposure. GO analysis shows that noise affects SNc primarily in protein folding, vesicle secretion, and neurotransmission. In previous research, aberrant protein folding has been shown to disrupt physiological function and signal transduction, leading to neuronal damage and death, thereby contributing to the pathogenesis of PD [55,56]. Heat shock proteins (HSPs) assist in protein folding as molecular chaperones, interacting with α-synuclein and potentially inducing neuronal apoptosis [57]. Transcriptomic analysis of PD patients reveals significantly elevated expression levels of multiple HSPs, including Hspa1a, compared to healthy controls [58]. RNA-Seq also indicates downregulation of several proteins, including RNA-binding motif 3 (Rbm3). As an RNA-binding protein, RBM3 enhances neural cell survival through multiple mechanisms: accelerating ribosome assembly, influencing microRNA biosynthesis, stabilizing mRNA structure, and enhancing global de novo protein synthesis [59]. Overexpression of Rbm3 has been shown to mitigate the impact of neurotoxins like rotenone and MPTP on cells [60,61]. Combined with our findings and prior research, noise-induced exacerbation of PD likely involves multiple genes. Assessing their roles could elucidate the molecular mechanisms underlying noise-induced PD exacerbation.

There are several limitations in this study. While the 6-OHDA model is widely used as an animal model of PD, it still has certain disadvantages. Although we used desipramine before injecting 6-OHDA, we still cannot rule out the possibility that 6-OHDA may cause damage to other types of neurons, leading to nonspecific effects. This study used RNA-Seq to analyze the gene expression pattern of SNc tissue without conducting single-cell sequencing analysis, which may have largely overlooked the molecular changes of SNc^DA^ neurons subpopulation. In the current study, we primarily focus on the role of the IC-SNc^DA^ circuit in environmental noise-induced motor deficits in early-stage PD mouse model. While these results do not fully exclude the influence of noise exposure-related stress on motor deficits, the necessity of IC-SNc^DA^ circuit was verified for mediating the noise exposure-induced motor deficits and SNc^DA^ neuronal loss. Moreover, our previous work revealed that projections from CeA to SNc^DA^ neurons contribute to chronic stress-induced vulnerability to MPTP in mice [31]. Taken together, both the IC-SNc^DA^ circuit and CeA-SNc^DA^ circuit may interact to drive the noise exposure-induced motor deficits and SNc^DA^ neuronal loss in PD animal model. Although our results demonstrate the key role of the IC-SNc^DA^ circuit in noise-induced PD, it is important to note that all experiments have been validated exclusively in animal models and thus require further validation through clinical trials.

Currently, there are many types of potential signaling pathways for PD treatment, such as α-synuclein, transcription factor EB, and autophagic pathways [62,63]. Additionally, compounds like mucuna pruriens, chlorogenic acid, and ursolic acid have shown neuroprotective effects on DA neurons loss in animal models of PD [64–66]. In this study, our findings indicate that acute noise exposure induces reversible motor deficits in early-stage PD mouse model, while chronic noise exposure leads to irreversible motor deficits and promotes the SNc^DA^ neurons susceptibility to 6-OHDA insult. Also, this process is modulated by VMAT2 at the IC-SNc^DA^ circuit. Our findings uncover a previously unappreciated role of the IC-SNc circuit in early-stage PD mice in response to environmental noise, which has significance for preventing the onset and progression of PD and highlights the need for environmental harmony to reduce neurodegeneration.

## Materials and methods

### Ethics statement

All experimental mice were housed in the Laboratory Animal Center of Huazhong University of Science and Technology. All animal procedures in this study were strictly conducted in accordance with the Biosafety Law of the People’s Republic of China, the Regulations on the Administration of Laboratory Animals, and relevant national standards. The experimental protocols were approved by the Institutional Animal Care and Use Committee of Huazhong University of Science and Technology (Approval Number: S1871).

### Animals

Adult C57BL/6J mice (male, 8 weeks old) were purchased from Beijing Vital River Laboratory Animal Technology Co., Beijing, China. All the mice were group-housed (4 mice in a homecage) in a 12-hour-light/12-hour-dark cycle and given standard food and water ad libitum. Adult TH-Cre transgenic mice (male, 8–10 weeks old) were obtained from Jackson Laboratory, CA, USA [67].

### Viral vectors

The virus: AAV9-Syn-Cre, AAV9-DIO-Synaptophysin-EGFP, AAV9-DIO-RVG, AAV9-DIO-TVA-EGFP, RV-ENVA-ΔG-DsRed, AAV9-TH-Cre, AAV9-DIO-ChR2-mCherry, AAV9-DIO-mCherry, AAV9-DIO-hM4Di-mCherry, AAV9-DIO-hM3Dq-mCherry, AAV9-DIO-GCaMP6m, AAV9-DIO-VMAT2-EGFP, AAV1-TH-Cre, AAV9-DIO-EGFP, AAV9-DIO-NpHR-mCherry, AAVretro-Syn-Cre, AAV9-Syn-A53T-αSynuclein and AAV9-Syn were purchased from BrainVTA, Wuhan, China. All the viruses were subdivided into aliquots and stored at −80 °C until use. The viral titers ranged from 3  to  8 × 10^12^ genome copies/ml.

### Stereotaxic surgery

Prior to virus injection, mice were anesthetized with isoflurane (RWD Life Technology Co., Shenzhen, China) at 3%–4% for induction and 1%–3% for maintenance before being positioned on a stereotaxic apparatus (RWD Life Technology Co., Shenzhen, China). Mice were given a small cranial opening (0.5 mm^2^), and the viruses were injected using a microinjection pump via a glass microtubule. After each injection, the syringe was left in place for 10 min to allow for viral spread. The coordinates for viral infusion into the STR were as follows: anterior–posterior (AP) from bregma: +0.5 mm; mediolateral (ML), ±2.0 mm; and dorsal–ventral (DV) from the dura: −3.0 mm; SNc were as follows: AP, −3.08 mm; ML: ±1.2 mm; and DV −4.0 mm and IC were as follows: AP: −5.4 mm; ML: ±1.0 mm; and DV: −2.0 mm.

For anterograde tracing, a mixture of AAV9-DIO-Synaptophysin-EGFP and AAV9-Syn-CRE (1:1, 120 nl total volume) was unilaterally injected into the IC of mice. Three weeks later, the mice were perfused, and their brains were sliced for imaging.

For retrograde monosynaptic tracing, a mixture of helper viruses (AAV9-DIO-RVG and AAV9-DIO-TVA-EGFP) was unilaterally co-injected into the SNc of TH-Cre mice (1:2, 60 nl total volume). Following 14 days of helper virus expression, the mice were re-anesthetized, and RV-ENVA-ΔG-DsRed (60 nl) was infused into the same site within the SNc. Subsequently, the mice were transcardially perfused for imaging 7–10 days after the infusion of RV-ENVA-ΔG-DsRed.

For fiber photometry recording, a mixture of AAV9-TH-Cre and AAV9-DIO-GCaMP6m (1:1, 120 nl total volume) was unilaterally injected into the SNc of mice; or mice were unilaterally injected with AAV9-TH-Cre and AAV9-DIO-iGluSnFR into the SNc. Subsequently, a ceramic fiber-optic cannula (200 μm core diameter, 0.37 NA, Inper Technology Co., Hangzhou, China) was surgically implanted 200 μm above the SNc after 7 days of viral expression.

To activate the IC-SNc^DA^ circuit via optogenetic stimulation, mice were injected with AAV1-TH-Cre in the IC, followed by injections of AAV9-DIO-ChR2-mCherry or AAV9-DIO-mCherry in the SNc, and then optical fibers were implanted in the SNc. In chemogenetic manipulation experiments, AAV1-TH-Cre was injected into the IC, and AAV9-DIO-hM3Dq-mCherry or AAV9-DIO-hM4Di-mCherry was injected into the SNc, while the control group received AAV9-DIO-mCherry; alternatively, AAVretro-Syn-Cre was injected into the SNc, and the IC was injected with AAV9-DIO-hM3Dq-mCherry or AAV9-DIO-hM4Di-mCherry.

For cannula implantation in SNc, we lowered the cannulas into the SNc. Once cannulas were lowered, we attached them to the skull with UV-curing epoxy and then a thick layer of dental adhesive. After waiting 5 min for this to dry, we applied a very small amount of rapid-curing epoxy to attach the cannulas even more firmly to the underlying adhesive. The cannulas of each mouse were flushed with saline regularly after implantation. For drug or vehicle delivery, the infusion rate was set as 100 nL/min.

### Subacute low-dose 6-OHDA model

The same methods employed in virus injections were used for the 6-OHDA injections. A total of 0.75 μl 6-OHDA (3 mg/ml, Sigma) was dissolved in sterile SAL with ascorbic acid (0.2%) and injected into the STR of mice. The control mice received 0.75 μl of the vehicle (SAL with 0.02% ascorbic acid). Prior to the 6-OHDA injections, the animals were premedicated with desipramine (25 mg/kg, Sigma) to enhance the selectivity of 6-OHDA-induced lesions. Mice were provided with clear water supplementation for 1 week after surgery. All experiments were conducted at least 7 days after the surgery.

### MPTP model

MPTP (25 mg/kg, i.p., Sigma) was dissolved in SAL and injected for 5 consecutive days. The control mice received equal SAL injection. All experiments were conducted at least 24 h after the last MPTP injection.

### A53T model

Mice were bilaterally injected AAV9-Syn-A53T-αSynuclein into SNc, while the control group received AAV9-Syn injection. Forty days later, AAV9-retro-Syn-Cre was injected into the SNc of the mice, and AAV9-DIO-h4MDi-mCherry was injected into the IC.

### Acute or chronic noise exposure

The mice were placed in a soundproof chamber (interior size: 30 × 30 × 50 cm) with adequate ventilation in two cages (4 mice per cage) and provided with food and water ad libitum. The noise was generated in Adobe Audition 3.2, amplified, and presented via a free-field speaker. The speaker was placed above the mice cages. In the acute model, mice received 1-hour exposure to 85–100 dB SPL white noise after 7 days of 6-OHDA injection, while in the chronic model, they were exposed to the same noise 1 h per day for 7 consecutive days. All noises are randomly arranged, with a random interval of 5–30 s between each segment of noise. Each noise pulse had a variable duration ranging between 5 and 30 s, with the duration randomly selected for each segment. The intensity of the noise was randomized within 85–100 dB SPL across segments; for example, one segment might be set to 90 dB, while the next could be 95 dB, ensuring unpredictability in sound levels. Given the randomized inter-pulse intervals (5–30 s), the number of noise pulses per minute ranged from 2 to 12 pulses. Subsequent to noise exposure, the mice were returned to the animal breeding room, while control mice underwent identical procedures in the soundproof chamber but without noise stimulation. The protocols for acute and chronic music exposure are consistent with those for noise exposure, we replaced noise exposure with the similar high-decibel music (85–100 dB).

### Locomotion test

The locomotion test was conducted in the open field apparatus. One day prior to the test, mice were pre-adapted to the field for 5 min. At the start of the test, mice were placed in the center of the field and recorded for 15 min using the camera-tracking system (Shanghai Xinruan Information Technology Co., Shanghai, China). The open field area was cleaned after each trial. The distance traveled in 15 min was subsequently analyzed.

### Rotarod test

Before the test, mice were placed on the stationary rod (diameter = 6 cm) for a 5-minute acclimation period (Shanghai Xinruan Information Technology Co., Shanghai, China). Subsequently, all mice underwent training consisting of 3 rounds on the stationary rod, with speeds increasing from 5 to 10 rpm/min over 300 s. On the test day, all mice underwent three sessions with a 30-minute intersession interval. During each testing session, mice were placed on the rotating rod, which accelerated from 4 to 40 rpm over 300 s. The total time until the mice either fell off the rod or clung to it and circled around twice was recorded. Mice that remained on the rod for more than 300 s were manually removed, and their total time was recorded as 300 s. Following the completion of each session, the apparatus was cleaned using 75% ethanol. Finally, the average latency to fall was analyzed.

### Gait test

All gait analysis recordings were conducted using the VisuGait system (Shanghai Xinruan Information Technology Co., Shanghai, China). The system consists of a 130 cm tunnel elevated 152 cm above the ground. The tunnel features a glass floor with internal reflective green light and a ceiling containing red light to contrast with the internal reflective green light. A high-speed camera recorded the gait of the mice at a rate of 120 frames per second.

All mice underwent training for 2 days prior to formal experimentation. Training involved guiding each mouse through the tunnel until they could successfully traverse the platform uninterrupted. Any mouse that turned around or lingered on the glass platform for more than 30 s was immediately removed from the tunnel and allowed to begin a new crossing attempt. During formal experimentation, each mouse was allowed a maximum of 15 consecutive attempts to achieve successful passage twice. A trial (or run) was considered successful if it lasted within 4 s and was conducted at a steady pace (<100% speed variation). Following the completion of each session, the tunnel was cleaned using 10% ethanol.

### Immunofluorescence staining

In this experiment, mice were first anesthetized and then their hearts were perfused with a solution containing 4% paraformaldehyde. The brains were then fixed overnight at 4 °C. After that, coronal sections with a thickness of 35 μm were obtained using a Leica cryostat microtome following dehydration in sucrose solutions with concentrations of 20% and 30%. The brain slices were washed with PBST (1 × PBS containing 0.05% Tween 20), permeabilized with 0.3% Triton-X, and blocked with a solution containing 0.1% bovine serum albumin (BSA) and 3% goat serum in 1 × PBS for 1 h at 37 °C. Then, the brain slices were incubated separately with primary antibodies, including TH (1:1000, Proteintech) and c-FOS (1:1000, CST) at 4 °C for 16 h. After three rounds of washing with PBST for 5 min each, the brain slices were incubated with secondary antibodies for 90 min at 37 °C in the dark. The secondary antibodies used were goat anti-rabbit antibodies conjugated with Alexa Fluor 488 (Jackson ImmunoResearch, 1:200, green stain) or Alexa Fluor 594 (Jackson ImmunoResearch, 1:200, red stain). The cell nuclei were counterstained with DAPI, and the sections were mounted in fluorescent mounting medium and stored at 4 °C until analysis. Images were captured using an Olympus VS120 microscope and Zeiss LSM780.

### In vivo fiber photometry

The fiber-photometry system utilized in this investigation was provided by Inper Technology Co., Shenzhen, China. The 470 nm excitation wavelength was employed to induce fluorescence emission from the calcium indicator GCaMP6m, while a 410 nm excitation wavelength served as the internal control. Both wavelengths operated at a frequency of 15 Hz with an exposure time of 30 ms. The optical fiber was connected to the fiber photometry system and properly aligned to record the fluorescence signals during blue light stimulation. In these experiments, ΔF was defined as the difference between the recorded fluorescence transient and the basal F value. The basal F value was determined as the median fluorescence transients observed before the occurrence of significant events. All recordings were initiated once the photometry signal had stabilized. The GCaMP6m signals were quantified by normalizing the changes in their fluorescence signals (ΔF/F).

### In vivo optogenetic manipulation

For optogenetic inhibition of IC-SNc^DA^ circuit experiments, mice were subjected to yellow light (594 nm, 5–8 mW) stimulation on IC neurons while receiving noise exposure. For acute optogenetic activation of IC-SNc^DA^ circuit experiments, the output of nm blue light was measured by an optical power meter and adjusted to 4–6 mW (20 Hz, pulse width 10 ms). After a 5 min adaptation to a novel environment, mice received 10 min of bilateral continuous blue light stimulation, followed by immediate behavioral testing. The control group underwent the same stimulation protocol.

### In vivo chemogenetic manipulation

In the experiment investigating hM4Di-mediated inhibition of the IC-SNc^DA^ circuit under acute noise exposure conditions, C57BL/6J mice were divided into four groups: mCherry+SAL (*n* = 8), mCherry+CNO (*n* = 8), hM4Di+SAL (*n* = 8), and hM4Di+CNO (*n* = 7). Following 14 days of virus expression, 6-OHDA was administered at the injection site. Seven days later, mice received injections of SAL or CNO (1 mg/kg, i.p., Sigma) and underwent a 1-hour acute noise exposure 30 min later. Movement behaviors were evaluated thereafter.

For hM3Dq-mediated short-term stimulation experiments, C57BL/6J mice were divided into two groups: SAL (*n* = 8) and CNO (*n* = 8). Virus expression continued for 14 days, 6-OHDA was administered at the injection site. Seven days later, mice received SAL or CNO injections. After 90 min, mice were evaluated in locomotion, rotarod, and gait tests.

For the experiment examining hM3Dq-mediated chronic chemogenetic activation of the IC-SNc^DA^ circuit, another set of C57BL/6 mice were divided into four groups: mCherry+SAL (*n* = 8), mCherry+CNO (*n* = 8), hM3Dq+SAL (*n* = 8), and hM3Dq+CNO (*n* = 8). Virus expression continued for 14 days, followed by 6-OHDA injection at the same site. Seven days later, mice were administered SAL or CNO injections. After 7 consecutive days, mice underwent testing in locomotion, rotarod, and gait tests.

In the investigation of hM4Di-mediated inhibition of the IC-SNc^DA^ circuit in the chronic noise exposure model, C57BL/6 mice were divided into four groups: mCherry+SAL (*n* = 8), mCherry+CNO (*n* = 8), hM4Di+SAL (*n* = 8), and hM4Di+CNO (*n* = 8); or two groups: SAL (*n* = 8) and CNO (*n* = 8). Following 14 days of virus expression, 6-OHDA was administered at the injection site. Seven days later, mice received SAL or CNO injections and subsequently underwent 1-hour noise exposure daily for 7 days. Post-exposure, mice were evaluated in locomotion, rotarod, and gait tests.

In chronic IC-SNc^DA^ inhibition experiments, A53T and control mice were divided into four groups (Con+SAL, Con+CNO, A53T+SAL, A53T+CNO). Following 21-day viral expression, daily SAL or CNO injections and 1-hour noise exposure were administered for seven consecutive days, with motor behavioral assessments conducted on day 8.

### In vivo electrophysiological recordings

Broadband neural signals (0.3 Hz–7.5 kHz) were simultaneously recorded at 16 bits and 30 kHz from implanted 16-channel arrays using a multichannel data-acquisition system (Zeus, Bio-Signal Technologies, McKinney, TX, USA). Spikes were isolated using a high-pass filter set at 300 Hz, and real-time spike sorting was performed using principal component analysis. Subsequently, spike sorting refinement was carried out using Offline Sorter (Plexon, Dallas, TX, USA). Data analysis was conducted using NeuroExplorer 5 (Nex Technologies, Boston, MA, USA). The targeted brain regions for electrode implantation were specified as follows: SNc (AP: −3.08 mm; ML: +1.2 mm; DV: −4.0 mm). The electrodes were constructed using 16 individually insulated nichrome wires (35 μm inner diameter) with impedances ranging from 300 to 900 kΩ (Stablohm 675, California Fine Wire, USA). These wires were arranged in arrays of 16 micro-wires configured in a 3 × 5 × 5 × 3 pattern, with approximately 200 μm spacing between wires. The wires were connected to an 18-pin connector (Mil-Max), and the implanted electrodes were secured in place using dental cement. Based on the characteristic firing patterns of midbrain DA neurons, neurons with a baseline firing rate below 10 Hz and displaying long-duration action potentials (peak-to-peak duration > 450 ms) were identified as putative DA neurons.

### Image analysis

The imaging and cell counting procedures were carried out by experimenters who were unaware of the group assignments. Three consecutive immunofluorescence images (×10) of the SNc were captured using an automated scanning fluorescence microscope (Olympus, VS120). Cell counting was manually performed using the Cell Counter tool in ImageJ. This tool allows for counting multiple types of cells in the same image and saves parameters of the counting results for subsequent import and modification. Furthermore, to compare multiple specimens, staining, image acquisition (exposure time and gain), and image analysis were performed concurrently for the entire dataset. Unbiased stereological counting of TH neurons was performed as described elsewhere [68,69], stereological quantification of TH^+^ neurons was performed on six systematically random coronal sections spaced 240 µm apart, spanning the entire anteroposterior extent of the SNc. DA neurons were identified by their characteristic rounded/ovoid somata containing both DAPI-stained nuclei and cytoplasmic TH labeling. To perform TH stereological counting, we employed the following parameters: counting frame (50 × 50 µm), sampling grid (130 × 130 µm), and 13 µm optical dissector height.

### RNA-Seq

mRNA was isolated using magnetic beads coated with oligo and then fragmented into smaller pieces at an appropriate temperature using a fragment buffer. The first-strand cDNA was synthesized through random reverse transcription (primed by hexamers), followed by the synthesis of the second-strand cDNA. RNA Index Adapters and A-Tailing Mix were added and incubated to complete the repair process. PCR was conducted to amplify the cDNA fragments obtained in the previous step, and the amplified products were purified using Ampure XP Beads. These products were then dissolved in an EB solution and subjected to quality control analysis on the Agilent Technologies 2100 bioanalyzer. The double-stranded PCR products were heated, denatured, and circularized using a splint oligo sequence, resulting in the construction of a library with single-stranded circular DNA format. Amplification of the final library was carried out using phi29 to generate DNA nanoballs (DNBs), each containing over 300 copies of the same molecule. The DNBs were loaded onto the nanoarray, and single-end reads with 50 bases were produced using the BGIseq500 platform (BGI-Shenzhen, China).

SOAPnuke (v1.5.2) was used to filter the sequencing data. Subsequently, HISAT2 (v2.0.4) was used to map the reads to the reference genome. The reads were aligned to a gene set database constructed by BGI (Beijing Genomic Institute in Shenzhen) using Bowtie2 (v2.2.5). Gene expression levels were calculated using RSEM (v1.2.12). To visualize gene expressions across various samples, we employed pheatmap to generate heatmaps. Differential expression analysis was conducted using DESeq2 (v1.4.5). We filtered DEGs based on criteria of *P* < 0.05 and | log_2_(fold change) | > 0.4.

GO analysis was employed to elucidate genetic regulatory networks of interest by categorizing DEGs into hierarchical categories based on their involvement in biological processes, cellular components, and molecular functions.

The data that support the findings of this study have been deposited into CNGB Sequence Archive (CNSA) of China National GeneBank DataBase (CNGBdb) with accession number CNP0005849.

### Western blotting

The SNc tissues (approximately 0.5 mg) obtained from mice were lysed in RIPA buffer supplemented with a protease inhibitor cocktail. After lysis, the lysates were centrifuged at 12,000*g* for 15 min at 4 °C, and the resulting supernatants were utilized for immunoblotting on 10% gels. Subsequently, the proteins were transferred onto a polyvinylidene fluoride membrane. Following the blocking step with 5% nonfat dry milk in TBST for 3 h at room temperature, the blots were then incubated overnight at 4 °C with primary antibodies. The next day, the membranes were washed with TBST and incubated with horseradish peroxidase-conjugated secondary antibodies at room temperature for 1 h. After three washes with TBST, the antibody-reactive bands were visualized using enhanced chemiluminescence detection reagents (1:1, GE Healthcare) and a gel imaging system (Tanon, Shanghai, China). The primary antibodies used in assays were: VMAT2 (1:1000) and GAPDH (1:3000). Quantification was performed using the ImageJ software.

### Statistical analysis

All the results were presented as mean ± SEM with *P < 0.05, **P < 0.01, ***P < 0.001, and ns represents no significance. Statistical analyses included Mann–Whitney *U* test, Wilcoxon signed-rank test, unpaired *t* test, paired *t* test, and two-way ANOVA, where appropriate. All statistical analyses were performed using the GraphPad Prism software.

## Supporting information

S1 Fig Acute noise exposure induces motor deficits in MPTP model. **(A)** Experimental paradigm for establishing acute noise exposure in MPTP model and measuring movement behaviors at day 6 using locomotion test, rotarod test, and gait test. Saline+Control (Con) group (*n* = 8), Saline+Noise group (*n* = 8), MPTP+CON group (*n* = 7), MPTP+Noise group (*n* = 8). **(B–F)** Representative traces and statistics of mice in locomotion test (B, C), rotarod test (D), and gait test (E, F) on day 8. Data are presented as the mean ± SEM. *P < 0.05, **P < 0.01, ***P < 0.001, and ns for no significance. The data underlying this figure can be found in S8 Data.
(TIF)

S2 Fig Chronic noise exposure leads to motor deficits in MPTP mice. **(A)** The timeline of experimental scheme and diagram for chronic noise exposure in MPTP mice. *n* = 8 mice for each group. **(B–F)** Representative traces and statistics of mice in locomotion test (B, C), rotarod test (D), and gait test (E, F) on day 13. Data are presented as the mean ± SEM. *P < 0.05, **P < 0.01, ***P < 0.001, and ns for no significance. The data underlying this figure can be found in S9 Data.
(TIF)

S3 Fig Acute music exposure induces motor deficits in 6-OHDA model. **(A)** Experimental paradigm for establishing acute music exposure in 6-OHDA model and measuring movement behaviors at day 8 using locomotion test, rotarod test, and gait test. *n* = 8 mice for each group. **(B–F)** Representative traces and statistics of mice in locomotion test (B, C), rotarod test (D), and gait test (E, F) on day 8. **(G)** Representative images of anti-TH immunofluorescence in SNc and VTA. (F) Numbers of TH-neurons in the SNc and VTA were counted stereologically. *n* = 5 mice for each group. Data are presented as the mean ± SEM. *P < 0.05, **P < 0.01, ***P < 0.001, and ns for no significance. The data underlying this figure can be found in S10 Data.
(TIF)

S4 Fig Chronic music exposure leads to motor deficits and SNc^DA^ neuronal loss in 6-OHDA model. **(A)** The timeline of experimental scheme and diagram for chronic music exposure in 6-OHDA mice. *n* = 8 mice for each group. **(B–F)** Representative traces and statistics of mice in locomotion test (B, C), rotarod test (D), and gait test (E, F) on day 15. **(G)** Representative images of anti-TH immunofluorescence in SNc and VTA. (F) Stereological analysis was employed to estimate TH-neurons in the SNc and VTA. *n* = 5 mice for each group. Data are presented as the mean ± SEM. *P < 0.05, **P < 0.01, ***P < 0.001, and ns for no significance. The data underlying this figure can be found in S11 Data.
(TIF)

S5 Fig Noise exposure increased the firing frequency of SNc^DA^ neurons, whereas inhibition of the IC-SNc^DA^ circuit prevented this increase. **(A)** Experimental paradigm for in vivo electrophysiology recording in pre, noise, and noise+NpHR phase. **(B, C)** Representative raster plots (B) and statistical results depicting the firing rates of spontaneous spikes in putative SNc^DA^ neuron (C). Data are presented as the mean ± SEM. *P < 0.05, **P < 0.01, ***P < 0.001, and ns for no significance. The data underlying this figure can be found in S12 Data.
(TIF)

S6 Fig Direct monosynaptic projection from IC to SNc^DA^ neurons. **(A, B)** Representative fluorescence images of EGFP^+^ neurons in the IC and their terminal projections (green) to TH^+^ cells (red) in the SNc.
(TIF)

S7 Fig Direct monosynaptic projection from IC to SNc^DA^ neurons. **(A, B)** Fluorescence images showing starter cells in the SNc, co-infected with AAV9-DIO-RVG, AAV9-DIO-TVA-EGFP (green), and RV-ENVA-ΔG-DsRed (red) in a TH-Cre mouse; and the corresponding DsRed-labeled input neurons in the IC traced from these SNc DA starter cells.
(TIF)

S8 Fig IC neurons project to SNc^DA^ neurons. **(A)** Fluorescence images showing EGFP+ projections (green) and TH+ cells (red) in SNc. **(B)** Quantification of IC-derived synaptic terminals in the SNc^DA^ via stereology. *n* = 3 mice. **(C)** Fluorescence images of DsRed-labeled neurons in the IC traced from SNc^DA^ neurons. **(D)** Stereological quantification of IC-SNc^DA^ projection neurons. *n* = 3 mice. Data are presented as the mean ± SEM. *P < 0.05, **P < 0.01, ***P < 0.001, and ns for no significance. The data underlying this figure can be found in S13 Data.
(TIF)

S9 Fig IC neuron activation triggers a rapid increase in SNc^DA^ neuronal firing rates. **(A)** Experimental workflow for in vivo electrophysiological recording of SNc^DA^ neuronal firing rates during optogenetic activation of IC neurons. **(B, C)** Raster plots (B) and response latency analysis (C) of SNc^DA^ neuronal firing following 5 ms optogenetic activation of IC neurons. Data are presented as the mean ± SEM. *P < 0.05, **P < 0.01, ***P < 0.001, and ns for no significance. The data underlying this figure can be found in S14 Data.
(TIF)

S10 Fig Optogenetic activation of IC neurons enhances glutamate release in SNc^DA^ neurons, which is dependent on AMPA receptor. **(A)** Schematic diagram of viral injection, cannula drug delivery, and fluorescence recording. **(B)** Heatmaps illustrate the iGluSnFR fluorescence of the vehicle and NBQX groups in response to photostimulation of SNc^DA^ neurons. **(C)** Statistics results of peak and mean ΔF/F of fluorescence signals in the vehicle and NBQX group. Data are presented as the mean ± SEM. *P < 0.05, **P < 0.01, ***P < 0.001, and ns for no significance. The data underlying this figure can be found in S15 Data.
(TIF)

S11 Fig ChR2-mCherry expression was restricted to SNc^DA^ neurons. **(A)** Representative images of the viral injection sites in the SNc. Expression of AAV9-DIO-ChR2-mCherry is shown in red. SNc^DA^ neurons are labeled by anti-TH immunofluorescence (green).
(TIF)

S12 Fig Acute activation of IC-SNc circuit mimics motor deficits induced by acute noise exposure in 6-OHDA mice. **(A)** The timeline of experimental scheme and diagram for acute activation of IC-SNc circuit in 6-OHDA mice. *n* = 8 mice for each group. **(B–F)** Representative traces and statistics of mice in locomotion test (B, C), rotarod test (D), and gait test (E, F). **(G–I)** Representative images (G) and stereological statistics results of immunofluorescence with anti-TH in SNc (H) and VTA (I). *n* = 5 mice for each group. Data are presented as the mean ± SEM. *P < 0.05, **P < 0.01, ***P < 0.001, and ns for no significance. The data underlying this figure can be found in S16 Data.
(TIF)

S13 Fig Long-term inhibition of the IC-SNc circuit rescues chronic noise exposure-induced motor deficits and SNc^DA^ neuronal loss in 6-OHDA mice. **(A)** Experimental paradigm for chronic inhibition of IC-SNc circuit. *n* = 8 mice for each group. **(B–F)** Representative traces and statistics results of locomotion test (B, C), rotarod test (D), and gait test (E, F). **(G–I)** Representative anti-TH immunofluorescence images (G) and stereological quantification of TH-neurons in the SNc (H) and VTA (I). *n* = 5 mice for each group. *n* = 5 mice for each group. Data are presented as the mean ± SEM. *P < 0.05, **P < 0.01, ***P < 0.001, and ns for no significance. The data underlying this figure can be found in S17 Data.
(TIF)

S14 Fig Inhibition of the IC-SNc circuit during chronic noise exposure ameliorates motor deficits and reduces SNc^DA^ neuronal loss caused by chronic noise exposure in A53T mice. **(A)** Schematic diagram illustrating the establishment of the A53T α-synuclein mouse model and inhibition of the IC-SNc circuit. *n* = 7 mice for each group. **(B–F)** Representative traces and statistics results of locomotion test (B, C), rotarod test (D), and gait test (E, F). **(G–I)** Representative traces (G) and stereological statistics results of immunofluorescence with anti-TH in SNc (H) and VTA (I). *n* = 5 mice for each group. Data are presented as the mean ± SEM. *P < 0.05, **P < 0.01, ***P < 0.001, and ns for no significance. The data underlying this figure can be found in S18 Data.
(TIF)

S15 Fig No significant loss of SNc DA neurons was observed following two rounds of AAV9 administration. **(A)** Schematic of the viral injection strategy. *n* = 5 mice for each group. **(B–D)** Representative traces (B) and stereological statistics results of immunofluorescence with anti-TH in SNc (C) and VTA (D). *n* = 5 mice for each group. Data are presented as the mean ± SEM. *P < 0.05, **P < 0.01, ***P < 0.001, and ns for no significance. The data underlying this figure can be found in S19 Data.
(TIF)

S1 Data The data supporting the graphs shown in the Fig 1. (XLSX)

S2 Data The data supporting the graphs shown in the Fig 2. (XLSX)

S3 Data The data supporting the graphs shown in the Fig 3. (XLSX)

S4 Data The data supporting the graphs shown in the Fig 4. (XLSX)

S5 Data The data supporting the graphs shown in the Fig 5. (XLSX)

S6 Data The data supporting the graphs shown in the Fig 6. (XLSX)

S7 Data The data supporting the graphs shown in the Fig 7. (XLSX)

S8 Data The data supporting the graphs shown in the S1 Fig. (XLSX)

S9 Data The data supporting the graphs shown in the S2 Fig. (XLSX)

S10 Data The data supporting the graphs shown in the S3 Fig. (XLSX)

S11 Data The data supporting the graphs shown in the S4 Fig. (XLSX)

S12 Data The data supporting the graphs shown in the S5 Fig. (XLSX)

S13 Data The data supporting the graphs shown in the S8 Fig. (XLSX)

S14 Data The data supporting the graphs shown in the S9 Fig. (XLSX)

S15 Data The data supporting the graphs shown in the S10 Fig. (XLSX)

S16 Data The data supporting the graphs shown in the S12 Fig. (XLSX)

S17 Data The data supporting the graphs shown in the S13 Fig. (XLSX)

S18 Data The data supporting the graphs shown in the S14 Fig. (XLSX)

S19 Data The data supporting the graphs shown in the S15 Fig. (XLSX)

S1 Raw Images Western blot images for Figs 6I, 6L, 7D and 7O. (PDF)

## Abbreviations

6-OHDA: 6-hydroxydopamine
AP: anterior–posterior
CeA: central amygdala
DA: dopaminergic
DEG: differentially expressed gene
DNB: DNA nanoball
DV: dorsal–ventral
GO: Gene Ontology
HSP: heat shock protein
IC: inferior colliculus
ML: mediolateral
PD: Parkinson’s disease
Rbm3: RNA-binding motif 3
RNA-Seq: RNA sequencing
SAL: saline
SNc: substantia nigra compacta
SPL: sound pressure level
STR: dorsal striatum
TH: tyrosine hydroxylase
VMAT2: vesicular monoamine transporter 2
VTA: ventral tegmental area
