# Supplementary figures and images for: Environmental noise-induced changes to the IC-SNc circuit promotes motor deficits and neuronal vulnerability in a mouse model of Parkinson’s Disease
Source: PLoS Biol. 2025 Nov 4;23(11):e3003435. doi: 10.1371/journal.pbio.3003435 (PMC12585016; doi:10.1371/journal.pbio.3003435)

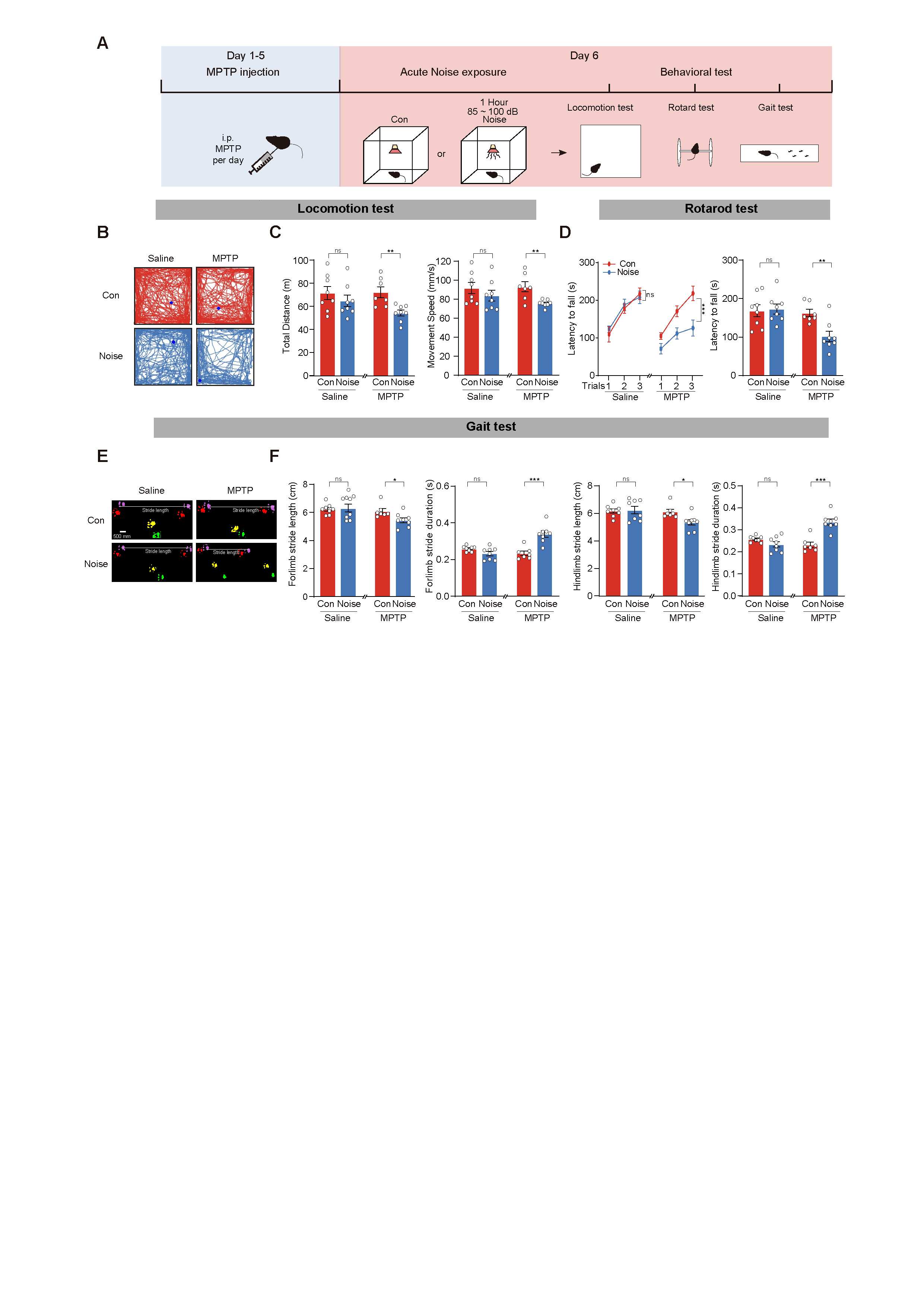

Supplement: S1 Fig — (A) Experimental paradigm for establishing acute noise exposure in MPTP model and measuring movement behaviors at day 6 using locomotion test, rotarod test, and gait test. Saline+Control (Con) group (n = 8), Saline+Noise group (n = 8), MPTP+CON group (n = 7), MPTP+Noise group (n = 8). (B–F) Representative traces and statistics of mice in locomotion test (B, C), rotarod test (D), and gait test (E, F) on day 8. Data are presented as the mean ± SEM. *P < 0.05, **P < 0.01, ***P < 0.001, and ns for no significance. The data underlying this figure can be found in S8 Data. (TIF) [file pbio.3003435.s001.tif]

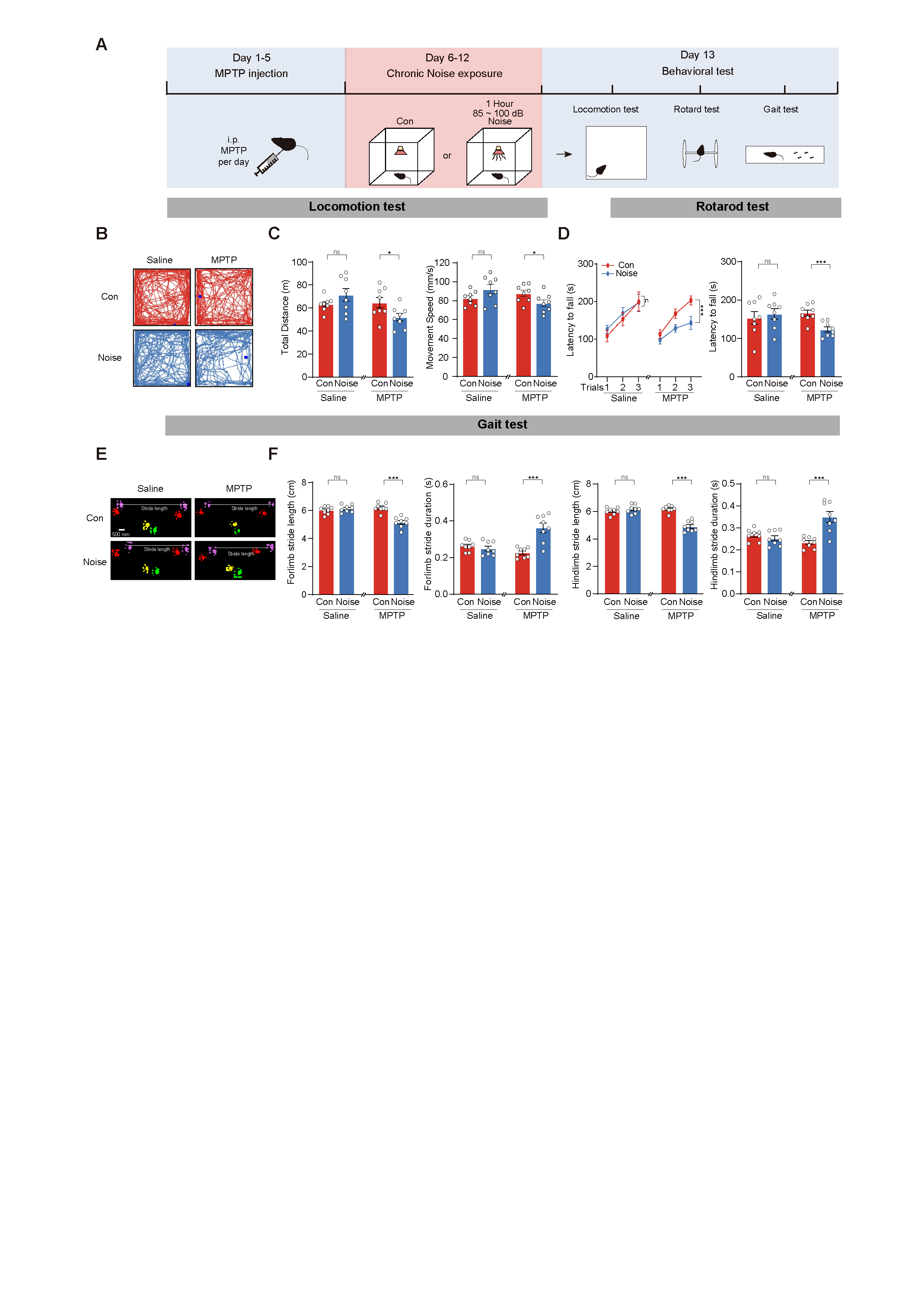

Supplement: S2 Fig — (A) The timeline of experimental scheme and diagram for chronic noise exposure in MPTP mice. n = 8 mice for each group. (B–F) Representative traces and statistics of mice in locomotion test (B, C), rotarod test (D), and gait test (E, F) on day 13. Data are presented as the mean ± SEM. *P < 0.05, **P < 0.01, ***P < 0.001, and ns for no significance. The data underlying this figure can be found in S9 Data. (TIF) [file pbio.3003435.s002.tif]

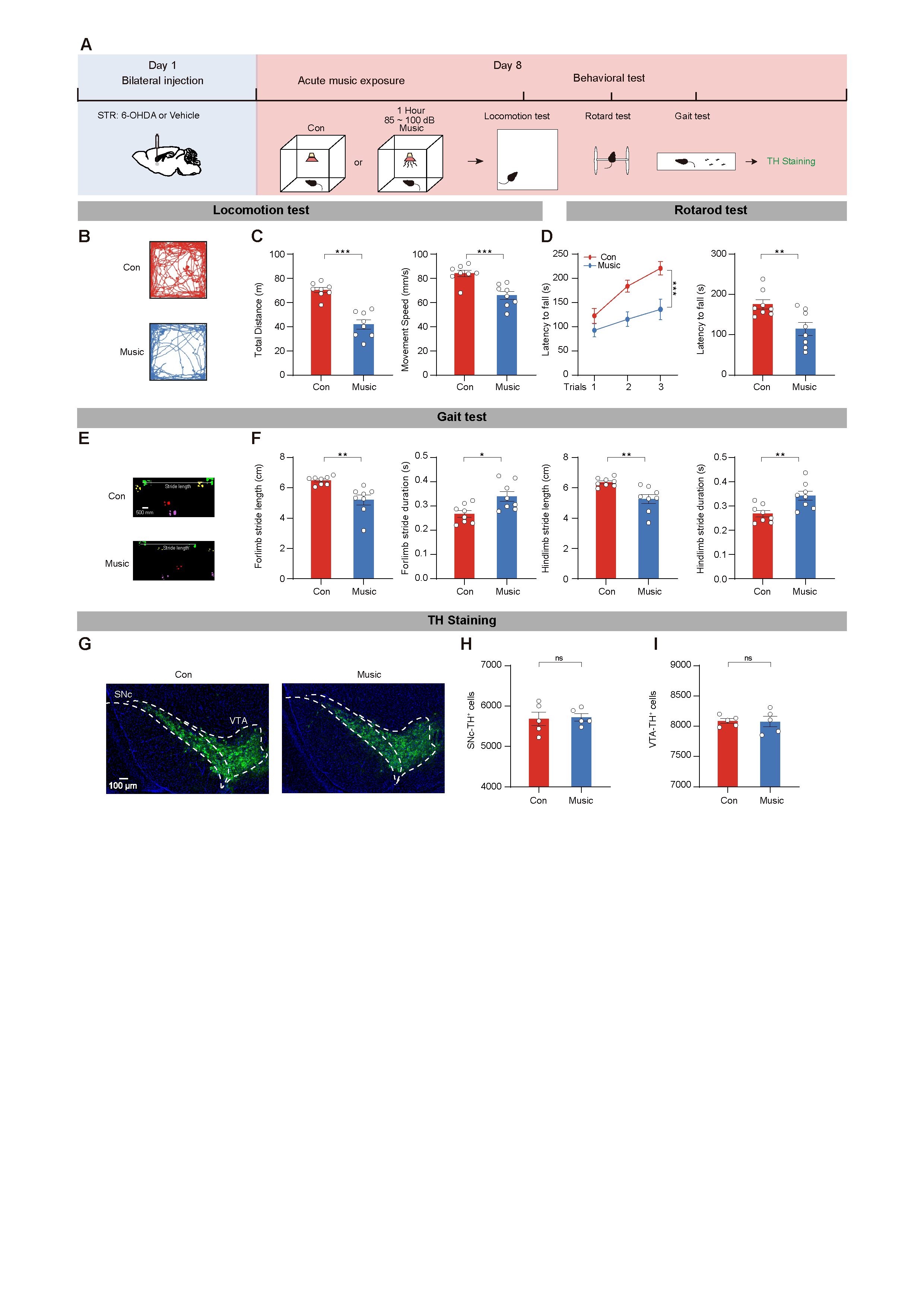

Supplement: S3 Fig — (A) Experimental paradigm for establishing acute music exposure in 6-OHDA model and measuring movement behaviors at day 8 using locomotion test, rotarod test, and gait test. n = 8 mice for each group. (B–F) Representative traces and statistics of mice in locomotion test (B, C), rotarod test (D), and gait test (E, F) on day 8. (G) Representative images of anti-TH immunofluorescence in SNc and VTA. (F) Numbers of TH-neurons in the SNc and VTA were counted stereologically. n = 5 mice for each group. Data are presented as the mean ± SEM. *P < 0.05, **P < 0.01, ***P < 0.001, and ns for no significance. The data underlying this figure can be found in S10 Data. (TIF) [file pbio.3003435.s003.tif]

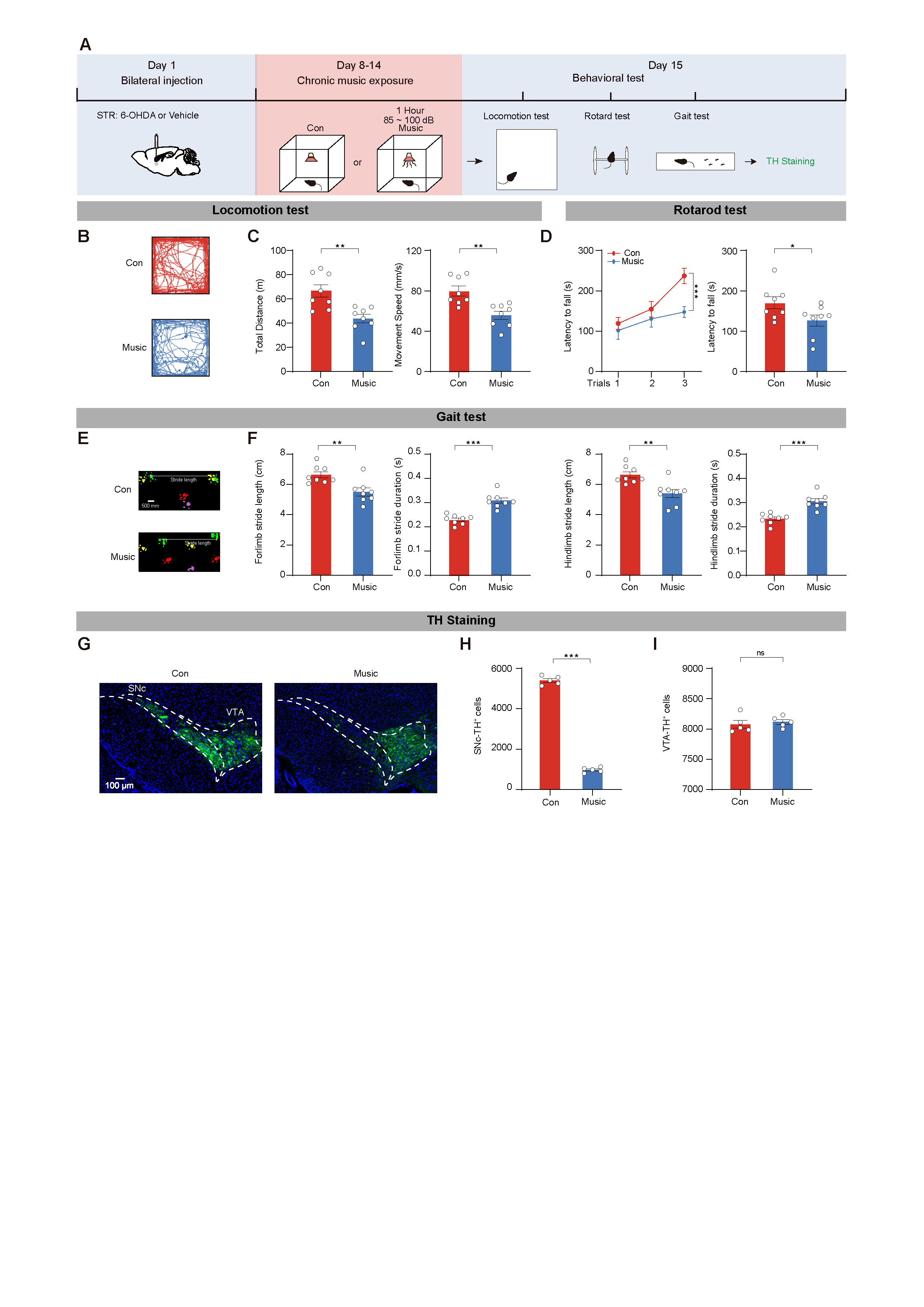

Supplement: S4 Fig — (A) The timeline of experimental scheme and diagram for chronic music exposure in 6-OHDA mice. n = 8 mice for each group. (B–F) Representative traces and statistics of mice in locomotion test (B, C), rotarod test (D), and gait test (E, F) on day 15. (G) Representative images of anti-TH immunofluorescence in SNc and VTA. (F) Stereological analysis was employed to estimate TH-neurons in the SNc and VTA. n = 5 mice for each group. Data are presented as the mean ± SEM. *P < 0.05, **P < 0.01, ***P < 0.001, and ns for no significance. The data underlying this figure can be found in S11 Data. (TIF) [file pbio.3003435.s004.tif]

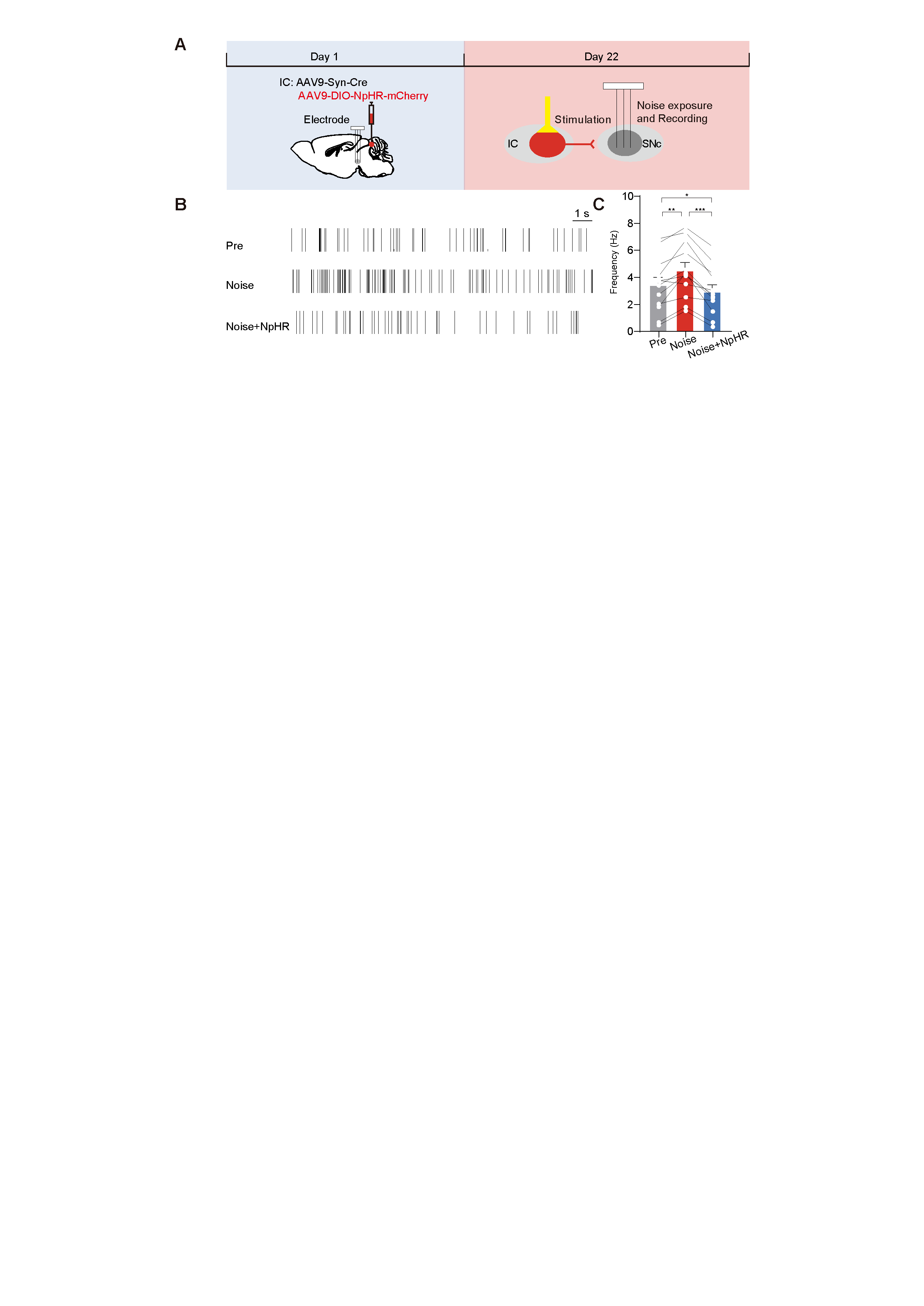

Supplement: S5 Fig — (A) Experimental paradigm for in vivo electrophysiology recording in pre, noise, and noise+NpHR phase. (B, C) Representative raster plots (B) and statistical results depicting the firing rates of spontaneous spikes in putative SNcDA neuron (C). Data are presented as the mean ± SEM. *P < 0.05, **P < 0.01, ***P < 0.001, and ns for no significance. The data underlying this figure can be found in S12 Data. (TIF) [file pbio.3003435.s005.tif]

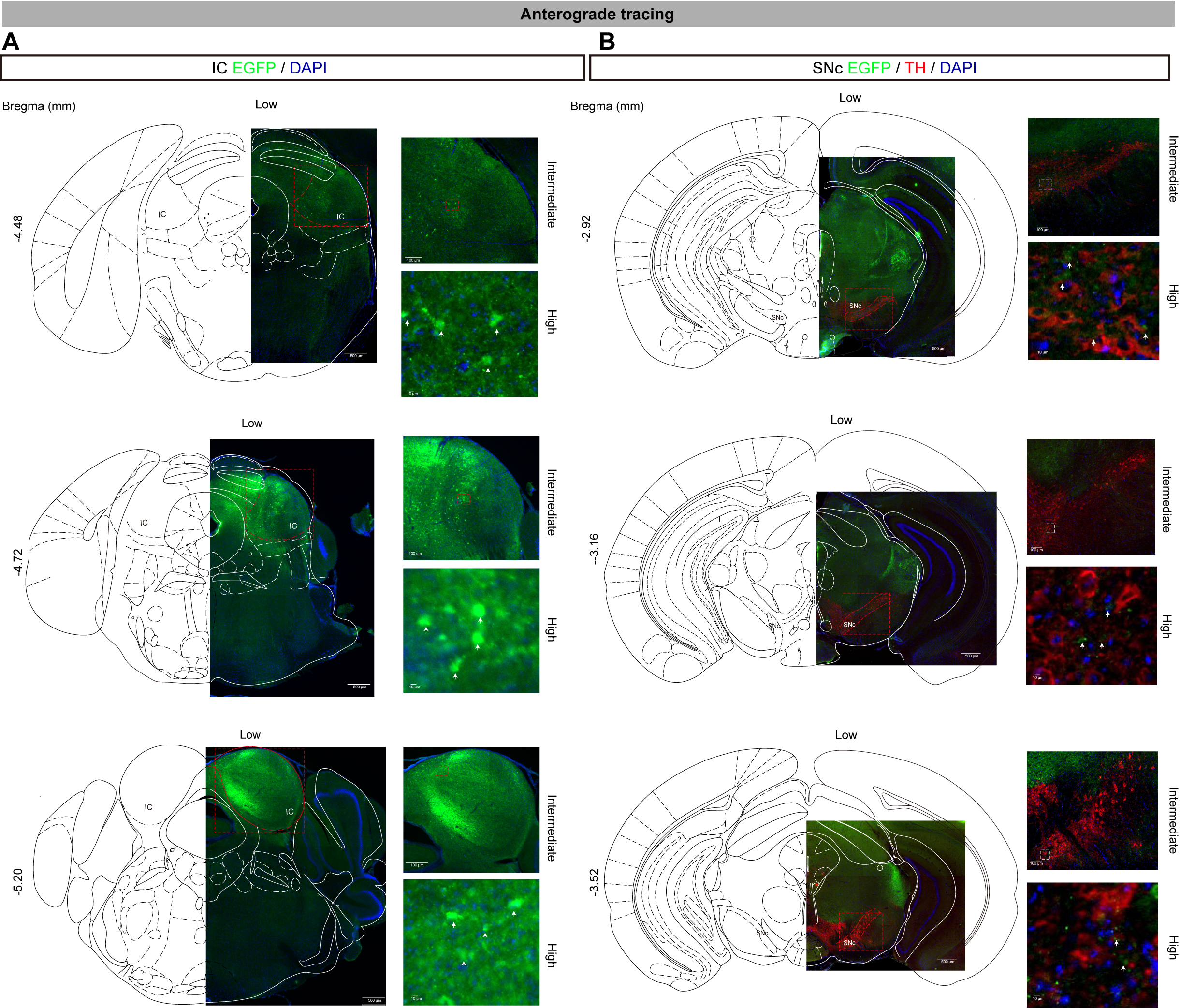

Supplement: S6 Fig — (A, B) Representative fluorescence images of EGFP+ neurons in the IC and their terminal projections (green) to TH+ cells (red) in the SNc. (TIF) [file pbio.3003435.s006.tif]

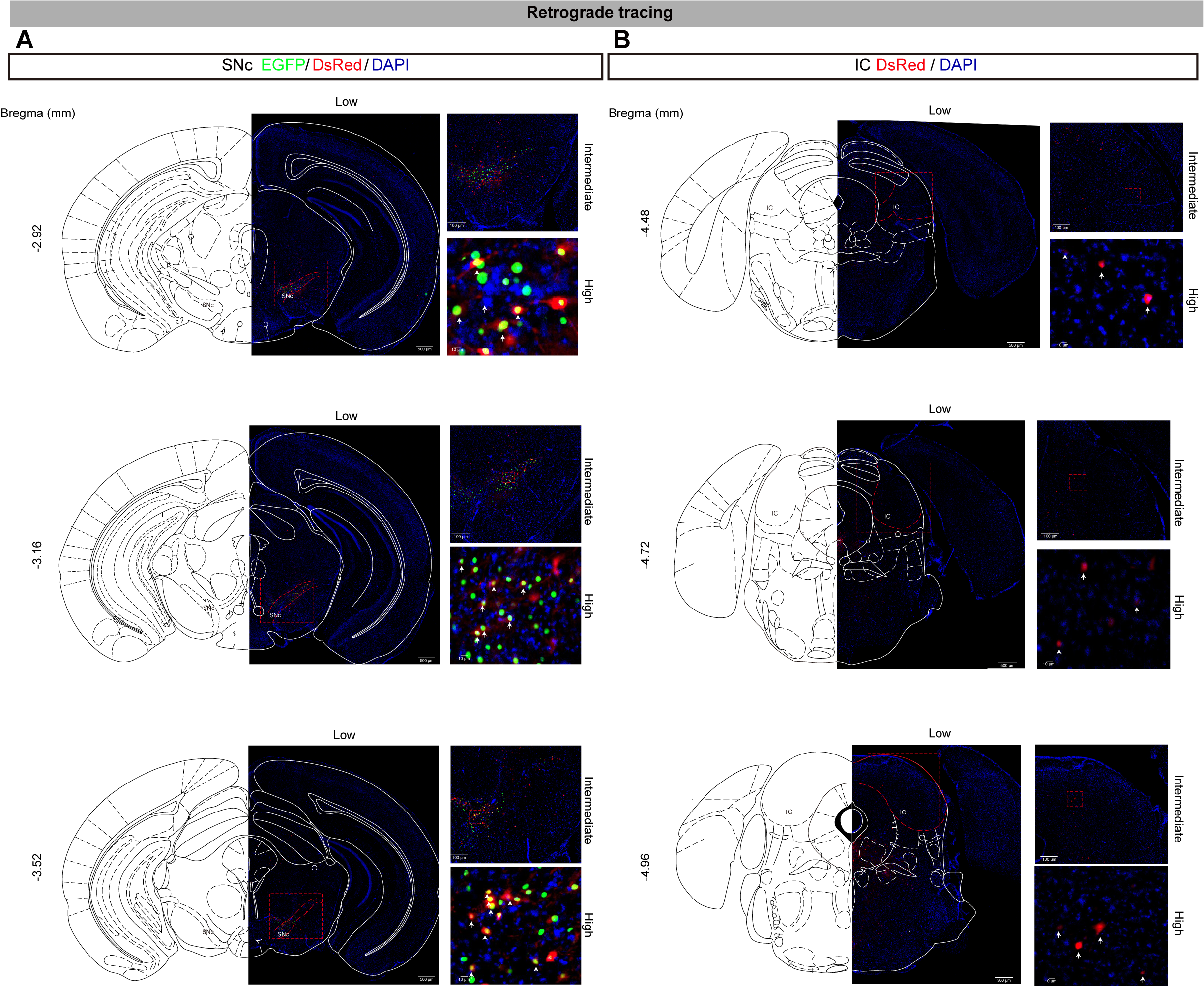

Supplement: S7 Fig — (A, B) Fluorescence images showing starter cells in the SNc, co-infected with AAV9-DIO-RVG, AAV9-DIO-TVA-EGFP (green), and RV-ENVA-ΔG-DsRed (red) in a TH-Cre mouse; and the corresponding DsRed-labeled input neurons in the IC traced from these SNc DA starter cells. (TIF) [file pbio.3003435.s007.tif]

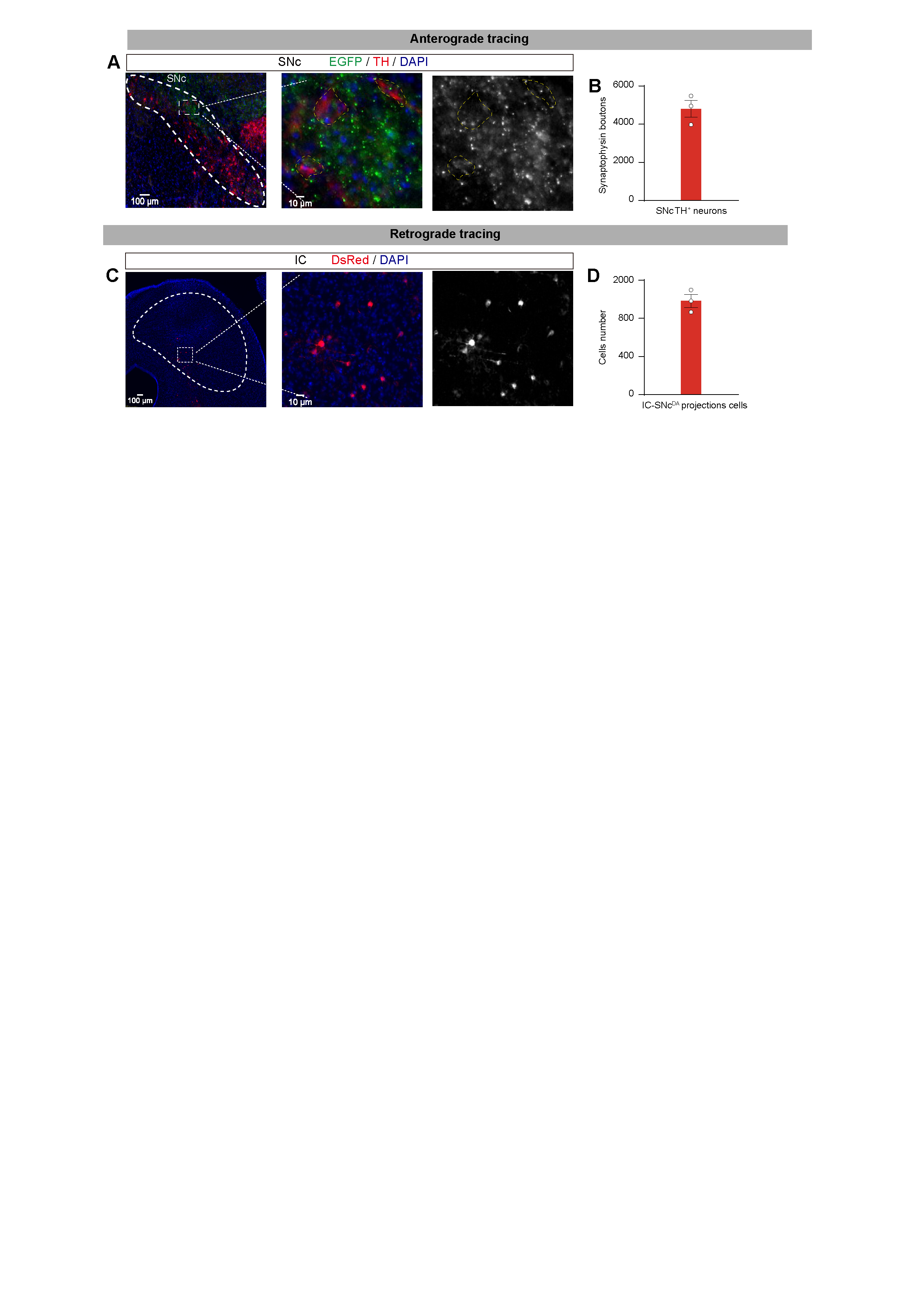

Supplement: S8 Fig — (A) Fluorescence images showing EGFP+ projections (green) and TH+ cells (red) in SNc. (B) Quantification of IC-derived synaptic terminals in the SNcDA via stereology. n = 3 mice. (C) Fluorescence images of DsRed-labeled neurons in the IC traced from SNcDA neurons. (D) Stereological quantification of IC-SNcDA projection neurons. n = 3 mice. Data are presented as the mean ± SEM. *P < 0.05, **P < 0.01, ***P < 0.001, and ns for no significance. The data underlying this figure can be found in S13 Data. (TIF) [file pbio.3003435.s008.tif]

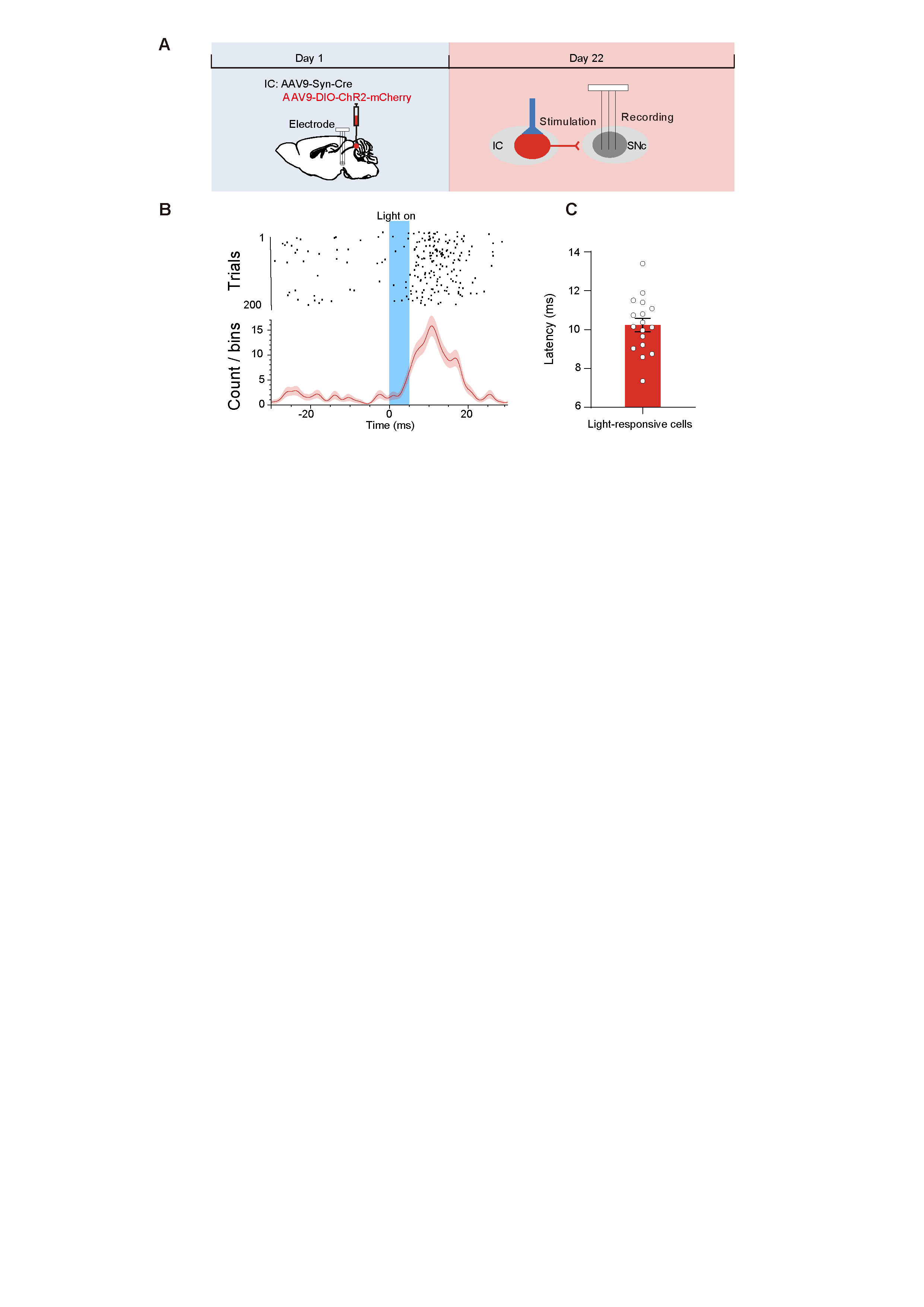

Supplement: S9 Fig — (A) Experimental workflow for in vivo electrophysiological recording of SNcDA neuronal firing rates during optogenetic activation of IC neurons. (B, C) Raster plots (B) and response latency analysis (C) of SNcDA neuronal firing following 5 ms optogenetic activation of IC neurons. Data are presented as the mean ± SEM. *P < 0.05, **P < 0.01, ***P < 0.001, and ns for no significance. The data underlying this figure can be found in S14 Data. (TIF) [file pbio.3003435.s009.tif]

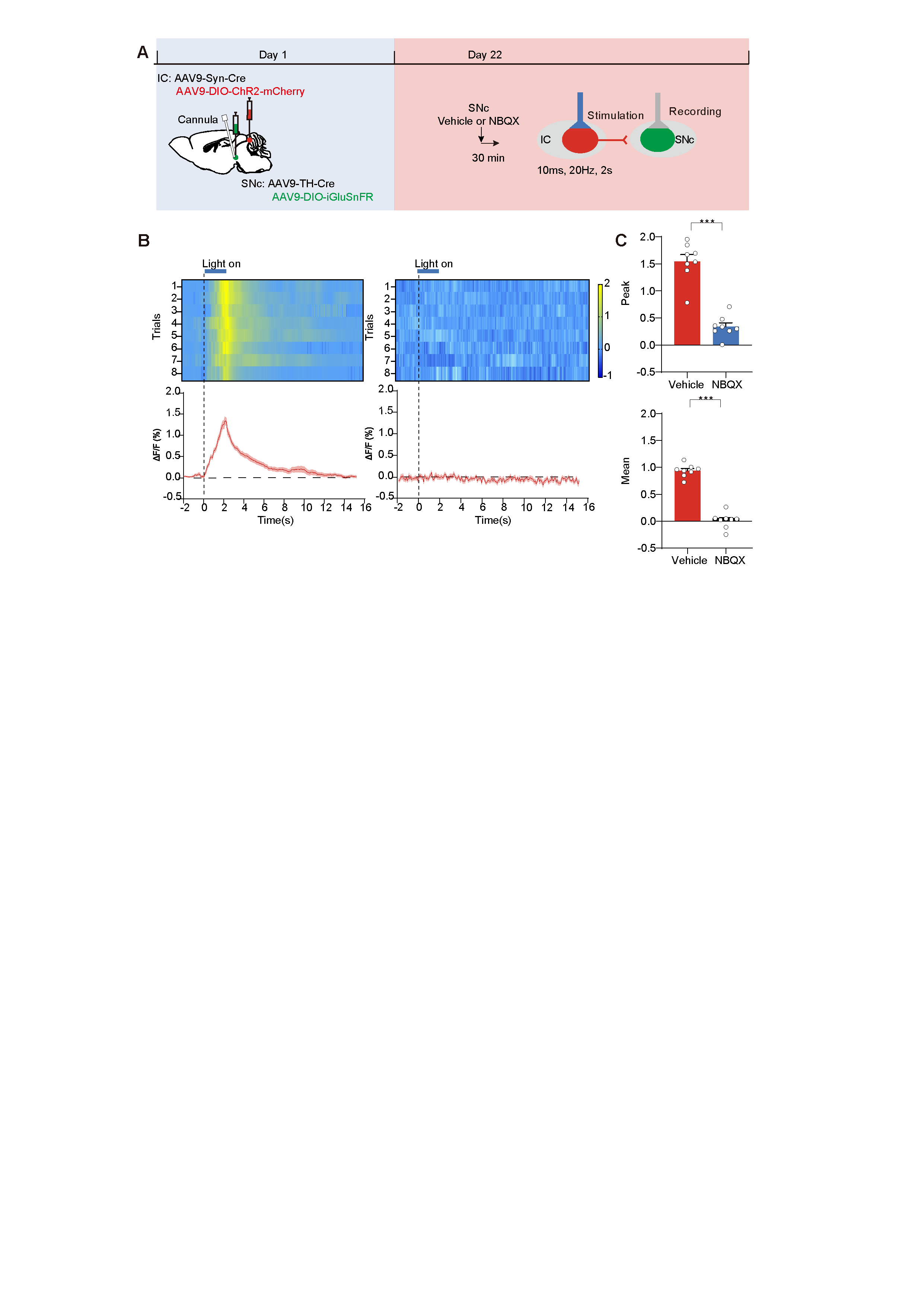

Supplement: S10 Fig — (A) Schematic diagram of viral injection, cannula drug delivery, and fluorescence recording. (B) Heatmaps illustrate the iGluSnFR fluorescence of the vehicle and NBQX groups in response to photostimulation of SNcDA neurons. (C) Statistics results of peak and mean ΔF/F of fluorescence signals in the vehicle and NBQX group. Data are presented as the mean ± SEM. *P < 0.05, **P < 0.01, ***P < 0.001, and ns for no significance. The data underlying this figure can be found in S15 Data. (TIF) [file pbio.3003435.s010.tif]

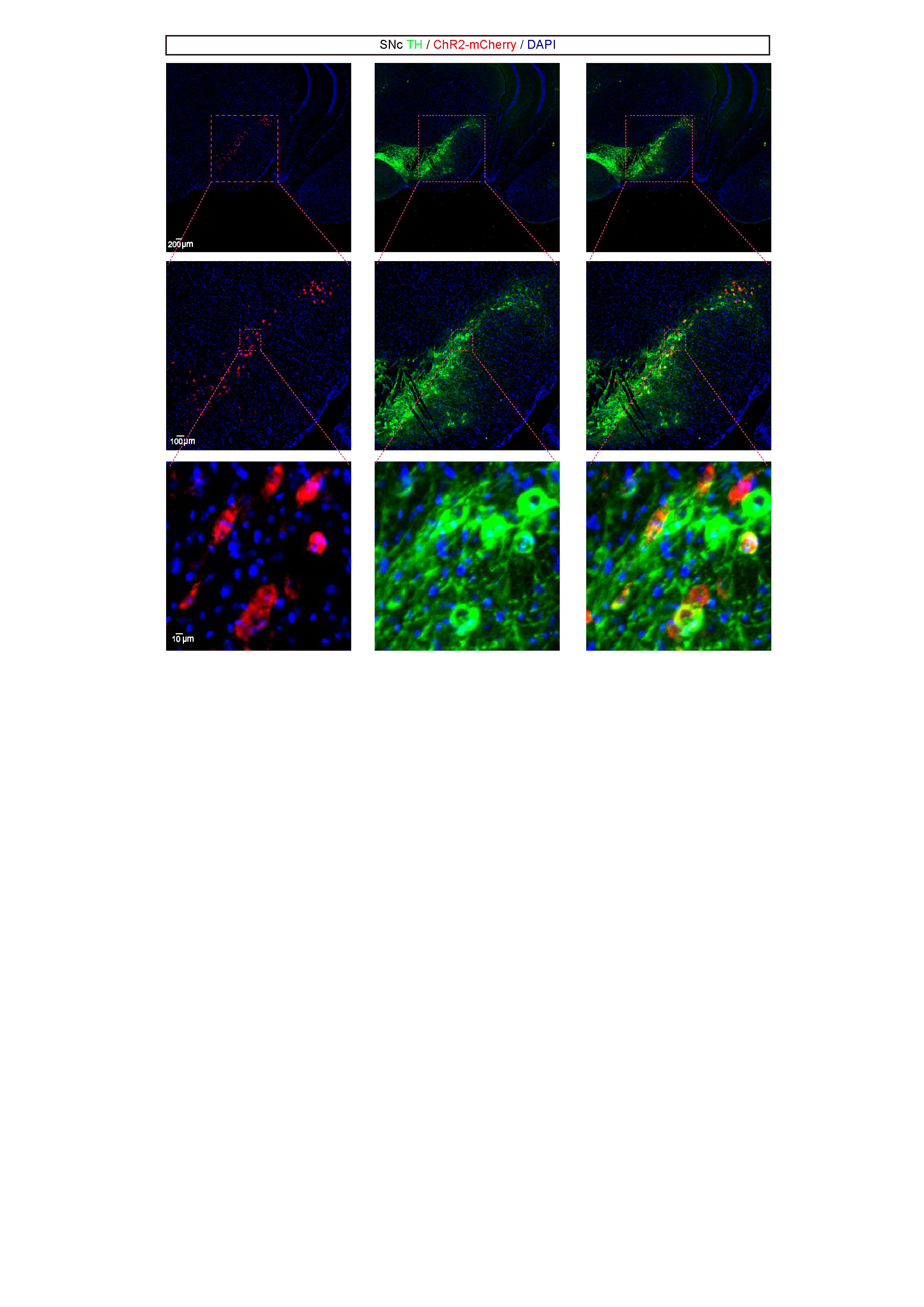

Supplement: S11 Fig — (A) Representative images of the viral injection sites in the SNc. Expression of AAV9-DIO-ChR2-mCherry is shown in red. SNcDA neurons are labeled by anti-TH immunofluorescence (green). (TIF) [file pbio.3003435.s011.tif]

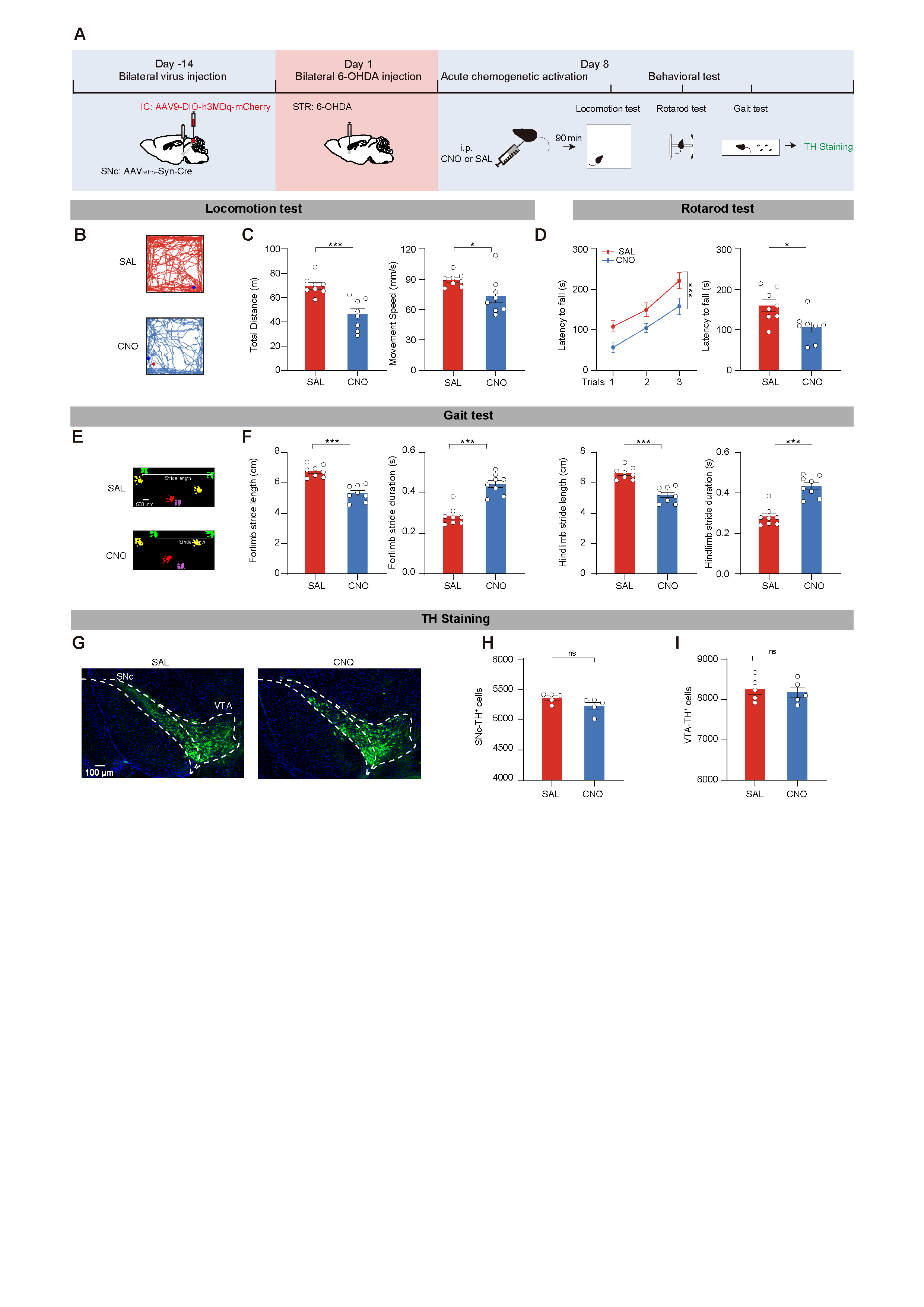

Supplement: S12 Fig — (A) The timeline of experimental scheme and diagram for acute activation of IC-SNc circuit in 6-OHDA mice. n = 8 mice for each group. (B–F) Representative traces and statistics of mice in locomotion test (B, C), rotarod test (D), and gait test (E, F). (G–I) Representative images (G) and stereological statistics results of immunofluorescence with anti-TH in SNc (H) and VTA (I). n = 5 mice for each group. Data are presented as the mean ± SEM. *P < 0.05, **P < 0.01, ***P < 0.001, and ns for no significance. The data underlying this figure can be found in S16 Data. (TIF) [file pbio.3003435.s012.tif]

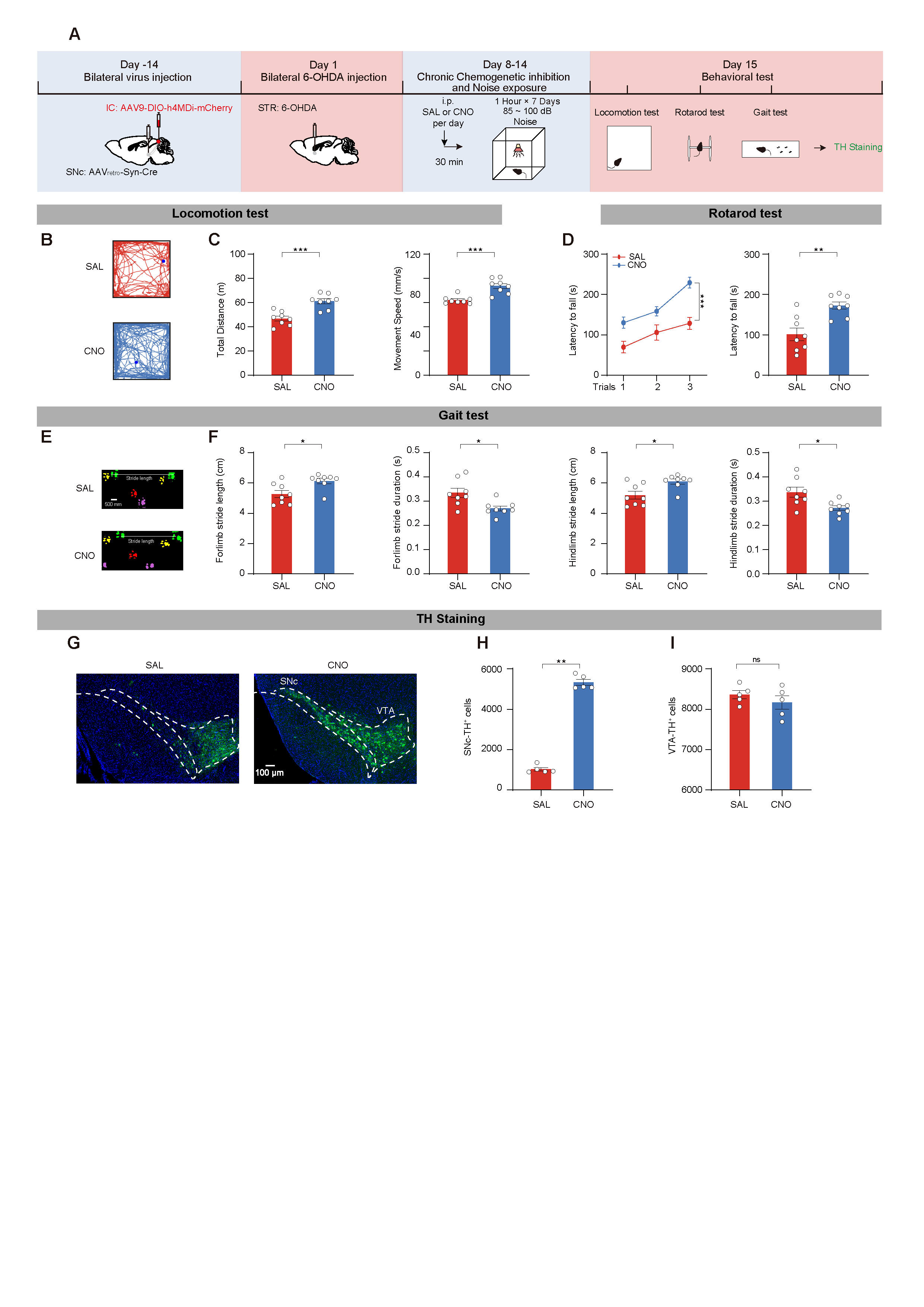

Supplement: S13 Fig — (A) Experimental paradigm for chronic inhibition of IC-SNc circuit. n = 8 mice for each group. (B–F) Representative traces and statistics results of locomotion test (B, C), rotarod test (D), and gait test (E, F). (G–I) Representative anti-TH immunofluorescence images (G) and stereological quantification of TH-neurons in the SNc (H) and VTA (I). n = 5 mice for each group. n = 5 mice for each group. Data are presented as the mean ± SEM. *P < 0.05, **P < 0.01, ***P < 0.001, and ns for no significance. The data underlying this figure can be found in S17 Data. (TIF) [file pbio.3003435.s013.tif]

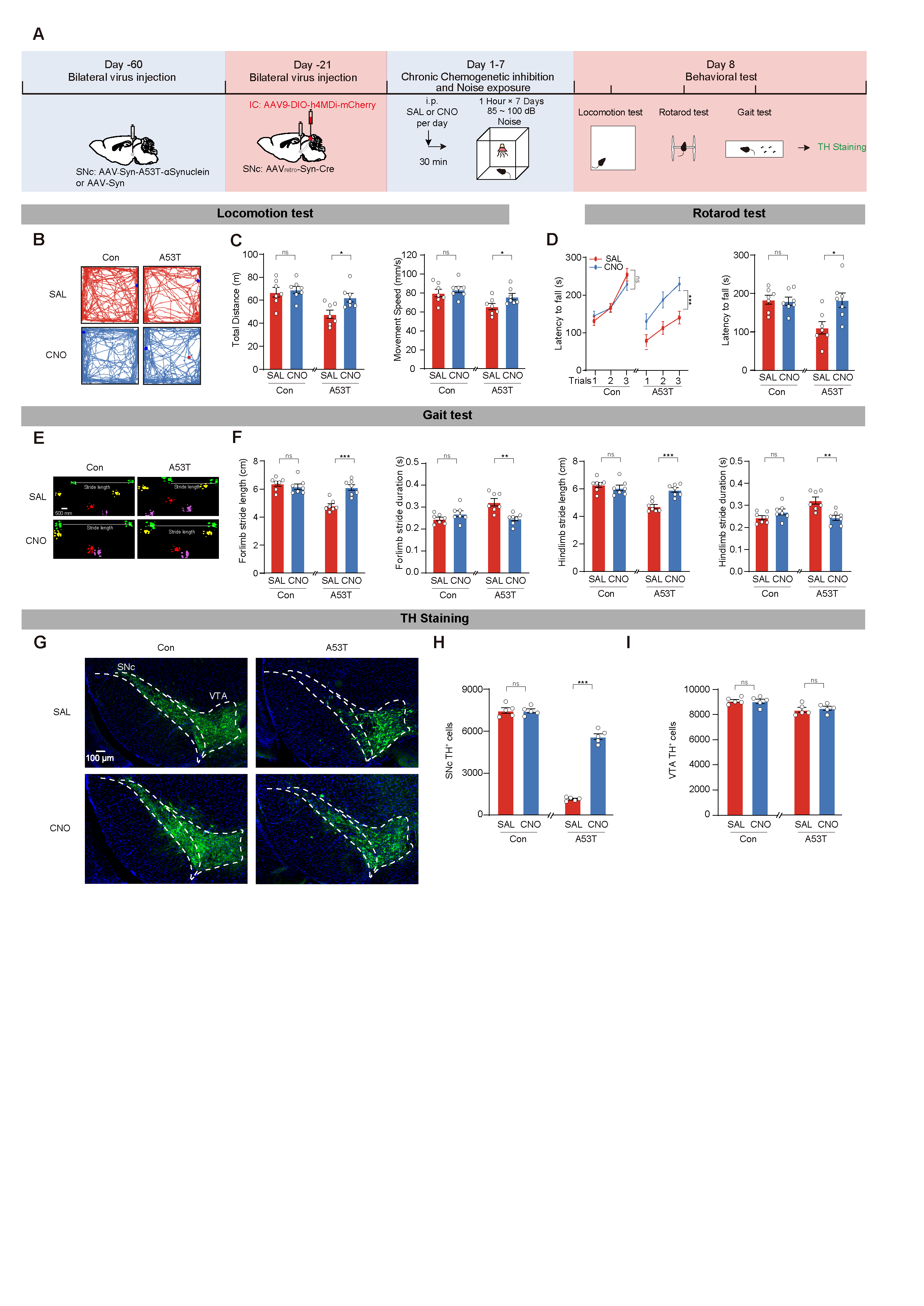

Supplement: S14 Fig — (A) Schematic diagram illustrating the establishment of the A53T α-synuclein mouse model and inhibition of the IC-SNc circuit. n = 7 mice for each group. (B–F) Representative traces and statistics results of locomotion test (B, C), rotarod test (D), and gait test (E, F). (G–I) Representative traces (G) and stereological statistics results of immunofluorescence with anti-TH in SNc (H) and VTA (I). n = 5 mice for each group. Data are presented as the mean ± SEM. *P < 0.05, **P < 0.01, ***P < 0.001, and ns for no significance. The data underlying this figure can be found in S18 Data. (TIF) [file pbio.3003435.s014.tif]

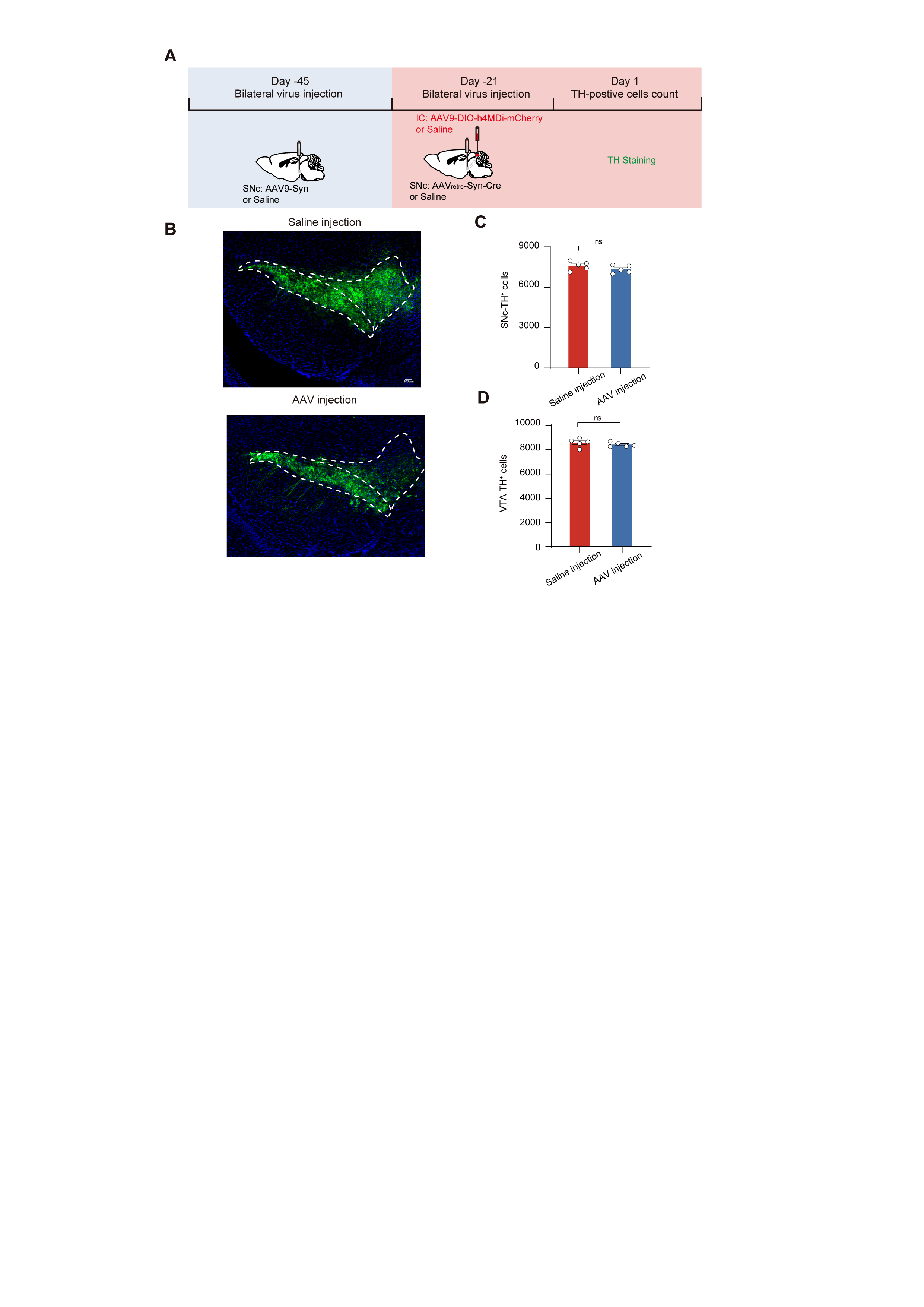

Supplement: S15 Fig — (A) Schematic of the viral injection strategy. n = 5 mice for each group. (B–D) Representative traces (B) and stereological statistics results of immunofluorescence with anti-TH in SNc (C) and VTA (D). n = 5 mice for each group. Data are presented as the mean ± SEM. *P < 0.05, **P < 0.01, ***P < 0.001, and ns for no significance. The data underlying this figure can be found in S19 Data. (TIF) [file pbio.3003435.s015.tif]
