# Supplementary material for: Environmental noise-induced changes to the IC-SNc circuit promotes motor deficits and neuronal vulnerability in a mouse model of Parkinson’s Disease
Source: PLoS Biol. 2025 Nov 4;23(11):e3003435. doi: 10.1371/journal.pbio.3003435 (PMC12585016; doi:10.1371/journal.pbio.3003435)
Supplement: S1 Raw Images — (PDF) [file pbio.3003435.s035.pdf]

Fig6_Raw_image

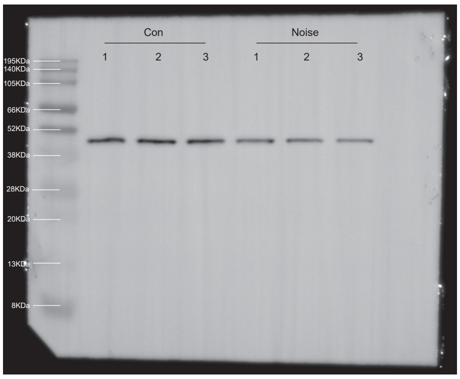

Fig. 6I VMAT2

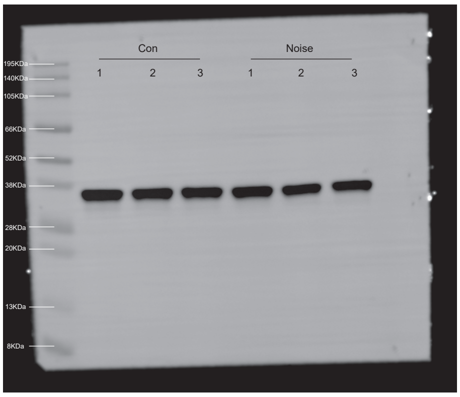

Fig. 6I GAPDH

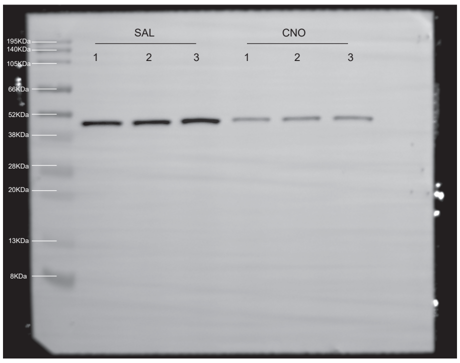

Fig. 6L VMAT2

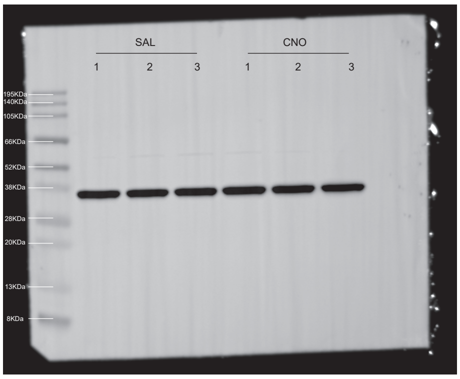

Fig. 6L GAPDH

Fig7_Raw_image

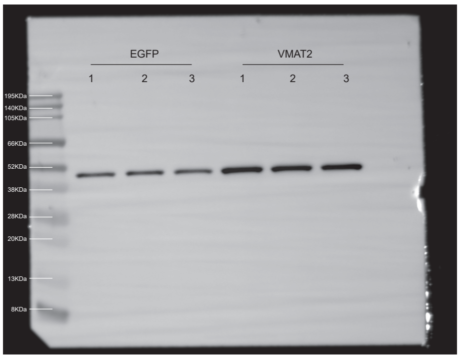

Fig. 7D VMAT2

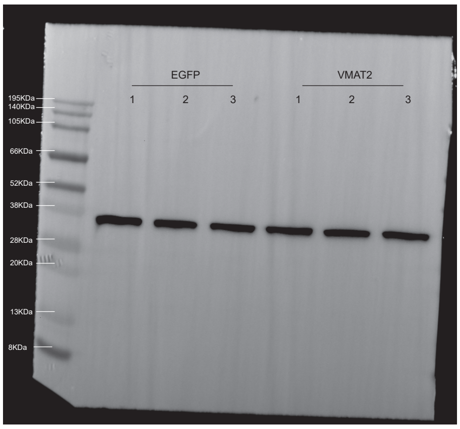

Fig. 7D GAPDH

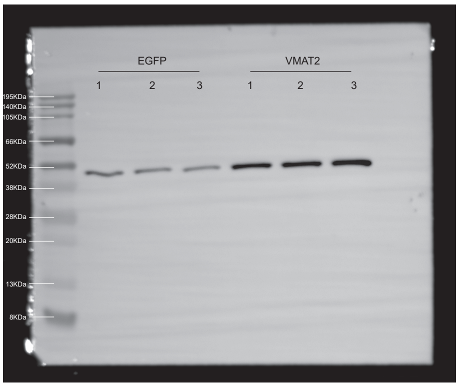

Fig. 7O VMAT2

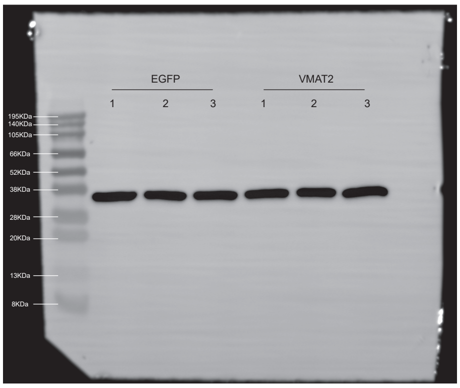

Fig. 7O GAPDH
